# Facet‐Dependent Water Inhibition of Alkanol Dehydration on TiO_2_ via Distinct Water–Alkanol Complexes

**DOI:** 10.1002/anie.2431054

**Published:** 2026-06-07

**Authors:** Wenda Hu, Haiting Cai, Anthony Savoy, Jinshu Tian, Sungmin Kim, Fan Lin, Junrui Li, Hao Xu, Yiqing Wu, Zihao Zhang, Nicholas Jaegers, Huamin Wang, Feng Gao, Jianzhi Hu, Yong Wang

**Affiliations:** ^1^ The Gene and Linda Voiland School of Chemical Engineering and Bioengineering Washington State University Pullman Washington USA; ^2^ Institute for Integrated Catalysis Pacific Northwest National Laboratory Richland Washington USA; ^3^ Zhejiang Key Laboratory of Surface and Interface Science and Engineering for Catalysts College of Chemical Engineering Zhejiang University of Technology Hangzhou Zhejiang China; ^4^ Center for Renewable Carbon School of Natural Resources University of Tennessee Knoxville Tennessee USA

**Keywords:** alkanol dehydration, facet‐dependent, TiO_2_ facet, water effect

## Abstract

Water is ubiquitous in biomass‐derived feeds, yet its molecular impact on oxygen‐elimination reactions remains poorly understood, particularly for catalysts exposing different facets. Here, we utilize well‐defined TiO_2_ nanocrystals with dominant (101) and (001) facets to reveal a pronounced facet‐dependent effect of water, where inhibition for dehydration of isopropanol (IPA) on the TiO_2_(001) surface is about four times more severe than TiO_2_(101). Through a combination of in situ solid state NMR, in situ infrared spectroscopy, kinetics studies, and theoretical calculations, we demonstrate that this disparity arises from the formation of distinct alkanol‐water complex intermediates. On TiO_2_(001), IPA undergoes dissociative adsorption to form an isopropoxide‐H_2_O complex that readily drives the surface into a complex‐dominated regime. This pathway increases the activation barrier for C–H cleavage by 40 kJ mol^−1^ by inducing a disordered transition state. In contrast, TiO_2_(101) favors molecular IPA adsorption with weak hydrogen bonding to water, resulting in a smaller complex formation constant and a much smaller activation barrier increase (25 kJ mol^−1^). By quantitatively linking facet‐dependent complex coverage to transition‐state destabilization, this work moves beyond simple site‐blocking models and provides a conceptual framework for designing catalysts that remain active in water‐containing environments.

## Graphical abstract

Water inhibits alkanol dehydration on TiO_2_ through facet‐dependent manners via distinct IPA‐water complexes. On TiO_2_(001), water forms a strongly hydrogen‐bonded isopropoxide–H_2_O complex that readily dominates the surface and substantially elevates the activation barrier, whereas on TiO_2_(101), water weakly hydrogen‐bonds to molecular IPA, resulting in a smaller complex formation constant and a much milder activation energy increase.

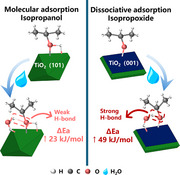

## 1 Introduction

Biomass upgrading is critical for producing renewable chemicals and fuels [1, 2, 3, 4]. Among various deoxygenation strategies, catalytic dehydration of biomass‐derived alcohols, polyols, and cycloalkanols is widely employed. Representative examples include fructose dehydration to hydroxymethylfurfural [5], dehydration of glycerol [6], and propene production from propanol [7]. However, a major challenge in these processes is the ubiquitous presence of water. Biomass feedstocks typically contain 6–95% water [8], and dehydration reactions inherently generate water as a product. Consequently, understanding how water influences alcohol dehydration is both fundamentally important and practically relevant for biomass conversion.

Water is widely recognized as a potent modulator of catalytic reactivity. In many oxide‐catalyzed systems, water often suppresses activity or accelerates deactivation [9, 10], although it can also enhance reactivity in specific systems such as alkane C–H activation [11], H–D exchange [12], and acid sites activation [13]. Mechanistically, water has been proposed to influence reactions through competitive adsorption, intermediate stabilization, or by forming unreactive species [14, 15]. Roy et al. [16] discovered that in ethanol and propanol dehydration on γ‐Al_2_O_3_, water competes for surface sites without altering activation barriers but adsorbs strongly enough to suppress alcohol adsorption. Zhi et al. [17] studied dehydration pathways of propanol on ZSM‑5 zeolites in the presence and absence of water and discovered an increase of activation barrier by forming a water‐propanol complex. Lin et al. [18] reported that the formation of bridging bidentate carboxylate associated with H_2_O leads to a higher ketonization activation barrier on TiO_2_. The formation of unreactive complexes, such as ethanol‐water heterodimers, was also observed in alcohol dehydration on Keggin‐type polyoxometalate clusters, γ‐Al_2_O_3_, and TiO_2_ [19, 20, 21, 22]. On Nb_2_O_5_, water dissociation generates Brønsted acid sites and decreases Lewis acidity, altering catalytic behavior [23]. In zeolites, Brønsted acid sites are solvated by water to form hydronium ions (H_3_O^+^), as shown by solid‐state NMR [24]. Bates et al. [25] revealed stabilization of (C_2_H_5_OH)(H_3_O^+^)(H_2_O)_4–5_ clusters in zeolite pores, which lowers the dehydration rate by decreasing the reaction order of ethanol.

Despite these insights, the molecular origins of water‐induced inhibition on heterogeneous oxide catalysts remain poorly understood. This gap arises from the lack of direct molecular‐level experimental evidence, and this elucidation is especially challenging because catalysts, for example, TiO_2_, expose multiple facets with distinct adsorption geometries and acid–base properties. TiO_2_ facets, such as the (101), (001), and (100) surfaces, exhibit markedly different surface energies (0.44, 0.90, and 0.53 J m^−2^, respectively) [26], leading to facet‐dependent reactivities in methanol decomposition, alkanol dehydration, and aldol condensation [27, 28, 29, 30, 31, 32, 33, 34]. Water–surface interactions also vary by facet: molecular water adsorbs preferentially on TiO_2_(101), while TiO_2_(001) favors dissociative adsorption at low coverages [35]. Therefore, these contrasts suggest that water may not affect dehydration uniformly across facets but rather interact with adsorbates in fundamentally different ways depending on the surface atomic arrangement. Recent advances have enabled facet engineering to expose well‐defined TiO_2_ facets, thereby reducing surface heterogeneity and offering a powerful platform to probe structure–reactivity relationships [36, 37, 38]. Despite this opportunity, a molecular‐level understanding of facet‐specific water effects under reaction‐relevant conditions is lacking. Most prior studies rely on either ultra‐high‐vacuum surface science or microscopic kinetic analysis, without directly correlating facet‐dependent adsorption structures to measured activation parameters. In particular, it remains unclear whether water simply reduces the number of available active sites or instead modifies the geometry, binding strength, and transition state through specific hydrogen‐bonding interactions with surface intermediates [17, 35, 39].

In this study, we address this question by systematically investigating how water modulates IPA dehydration on anatase TiO_2_ with (101) and (001) dominant facets, respectively, to unravel two distinct facet‐dependent water inhibition mechanisms at a molecular level. Compared to multifunctional substrates such as glycerol or sugars, which may undergo parallel reactions (e.g., rearrangement, C–C cleavage, oligomerization) that obscure surface chemistry, isopropanol (IPA) with a simple structure allows for direct probing of adsorption configurations, surface intermediates, and their interaction with co‐adsorbed water. Furthermore, as a well‐defined reaction involving the removal of one hydroxyl group and one H atom, using IPA dehydration as a probe reaction enables a clear and unambiguous investigation of how water influences reaction intermediates and activation energies on specific TiO_2_ facets. With a combination of transient and steady‐state reaction kinetics, including isotope labeling, in situ ^1^H‐^13^C cross polarization (CP) and direct polarization (DP) magic angle spinning (MAS) NMR, in situ IR spectroscopies, and density functional theory (DFT) calculations, we demonstrate that water inhibition is fundamentally facet‐dependent and not due to competitive adsorption. Instead, water reorganizes the adsorbate through the formation of distinct alcohol–water complexes depending on the underlying facet. On TiO_2_(001), water interacts strongly with dissociatively adsorbed isopropoxide to form a hydrogen‐bonded isopropoxide–H_2_O complex dominating on the surface that significantly perturbs adsorption geometry and increases the activation barrier for E2 elimination. In contrast, on TiO_2_(101), water interacts more weakly with molecularly adsorbed IPA, leading to a smaller complex formation constant and a more modest increase in activation barrier. This work establishes that water inhibition on oxide catalysts arises from facet‐specific solvation of surface intermediates. Beyond IPA dehydration, the resulting insights into facet‐dependent adsorption modes, hydrogen‐bonding environments, and associated enthalpic penalties reflect general principles governing alcohol dehydration under water‐containing conditions. These findings advance conventional site‐blocking descriptions and highlight the importance of facet control in dictating how interfacial hydrogen‐bond networks reshape catalysis under water‐rich conditions.

## 2 Results and Discussion

### 2.1 Physicochemical Properties of the Model Catalysts

**FIGURE 1:**
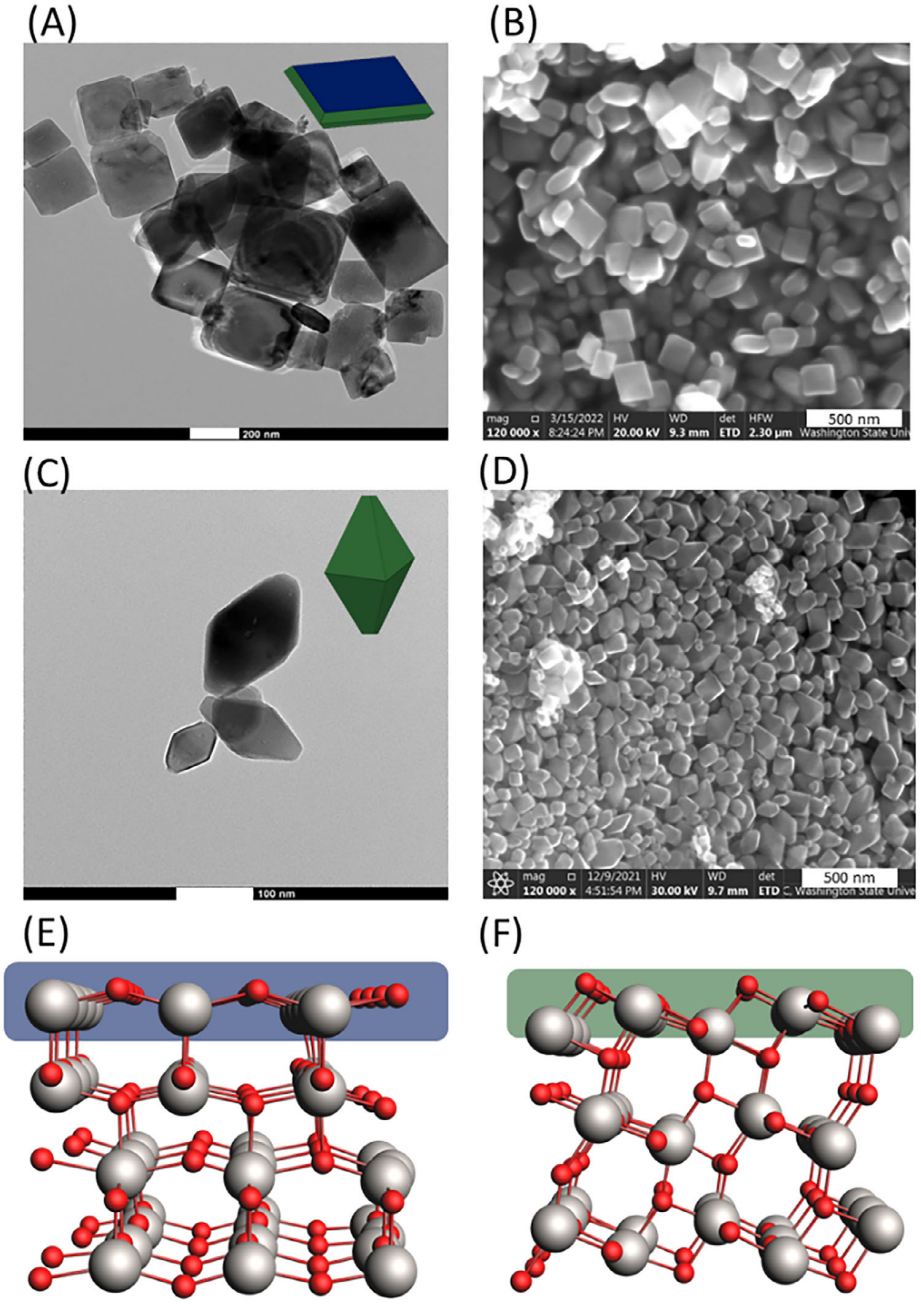
(A) and (B) TEM and SEM images of the TiO_2_(001); (C) and (D) TEM and SEM images of the TiO_2_(101); ball and stick models of (E) (001) and (F) (101).

TiO_2_(101) and (001) samples prepared by the two‐step hydrothermal synthesis exhibit distinct physicochemical properties [31, 32, 33, 40, 41, 42, 43]. The XRD patterns shown in Figure S1 demonstrate the anatase phase of synthesized TiO_2_(101) and (001). Figure 1A,B shows transmission electron microscopy (TEM) and scanning electron microscopy (SEM) images of the TiO_2_(001) sample, which exhibits a nanosheet morphology with an average lateral dimension of ∼300 nm. Facet analysis indicates ∼60% exposure of (001) facets (highlighted in blue in the inset model), with the remainder predominantly (101) facets (green), detailed in Supporting Information: Note 2, Figure S2, and Table S1. The sample has a surface area of 11 m^2^ g^−1^. Figure 1C,D presents TEM and SEM images of the TiO_2_(101) sample, displaying a bipyramidal morphology with an average particle size of ∼200 nm. Shape analysis reveals ∼93% exposure of (101) facets, with minor (001) contributions, mainly at particle tips. This sample exhibits a surface area of 17 m^2^ g^−1^. Post‐reaction analysis confirms that both the morphology and the relative (001)/(101) facet ratios are preserved within experimental uncertainty, indicating that the exposed TiO_2_ surfaces remain stable under reaction conditions (Figure S2C,D). Ball‐and‐stick models of the TiO_2_ surfaces are shown in Figure 1E,F. The (001) surface exhibits a relatively flat atomic arrangement, whereas the (101) surface displays a corrugated arrangement with alternating rows of Ti and O atoms. Densities of surface Lewis acid sites were determined by NH_3_‐temperature programed desorption (TPD). As shown in Figure S3A, NH_3_ desorbs at a relatively higher temperature from TiO_2_(001) than (101), consistent with a previous report [31]. The Lewis acid site density was calculated from NH_3_ uptake, yielding 21 and 14 µmol g^−1^ for TiO_2_(101) and (001), respectively, and was used for turnover frequency (TOF) normalization (Supporting Information, experimental section).

### 2.2 Kinetic Performance of Facet‐Dependent Water Impact on IPA Dehydration

**FIGURE 2:**
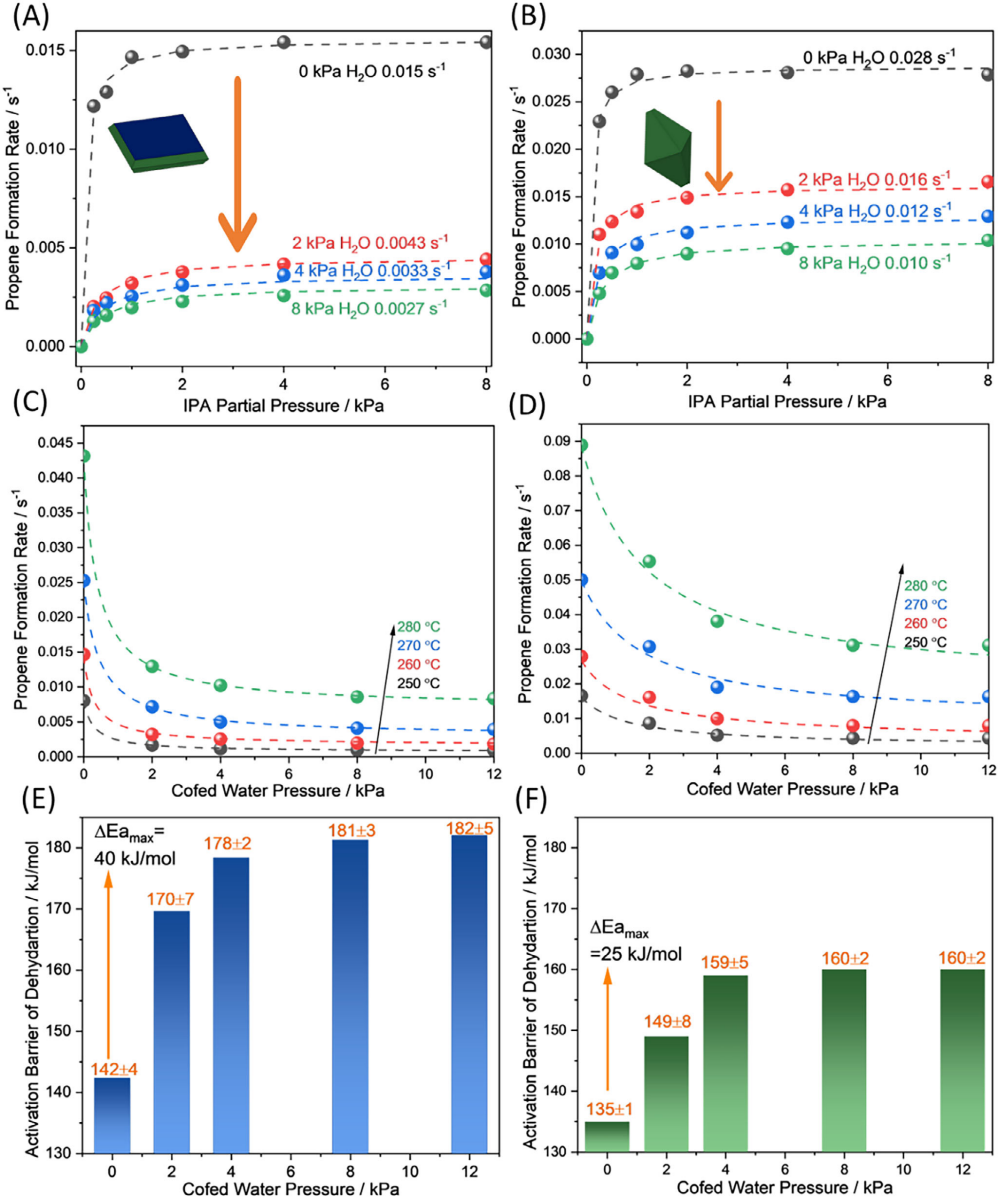
Kinetic dependences of propene formation rates over (A) TiO_2_(001) and (B) (101) on IPA and water pressures at 260°C; kinetic dependences of propene formation rates over (C) TiO_2_(001) and (D) (101) on water at 250°C–280°C and 1 kPa IPA; activation barriers on (E) TiO_2_(001) and (F) (101) at 0–12 kPa co‐fed water and 1 kPa IPA. Operating conditions: 250°C–280°C, 1 atm total pressure with 0.25–8 kPa IPA, 0–8 kPa H_2_O, and balance He, and 47.6 cm^3^ g^−1^ s^−1^ gas hourly space velocity (GHSV). Dashed lines in (A)—(D) show the rates calculated based on parameters obtained from non‐linear regression using Equation (1).

Figure 2 presents the kinetic dependences of steady‐state IPA dehydration on TiO_2_(101) and (001) at 250–280°C and an ambient pressure with 0.25–8 kPa IPA and 0–12 kPa H_2_O, where the conversions of IPA dehydration were below 10%. In the absence of co‐fed water, the propene formation rate at 260°C first increases and then plateaus with increasing IPA partial pressure on both TiO_2_(101) and (001) (Figure 2A,B), where the intrinsic activity on TiO_2_(101) is about two‐fold higher than that on TiO_2_(001), consistent with the prior report [31]. Introducing 2, 4, or 8 kPa of H_2_O into the feed of 8 kPa IPA, propene formation rates dramatically decreased by 3.5, 4.5, and 5.6 times, respectively, on TiO_2_(001), and by 1.7, 2.3, and 2.8 times, respectively, on TiO_2_(101). The larger rate drops on TiO_2_(001) indicate the substantially stronger sensitivity of TiO_2_(001) to water inhibition. Because the TiO_2_(001) sample exposes ∼60% (001) facets and ∼40% (101) facets, while TiO_2_(101) sample exposes ∼93% of (101) facets and ∼7% (001) facets, the measured rates reflect mixed‐facet behavior. By extrapolating the rate to 100% (101) and 100% (001) surfaces, detailed in Figure S4 and SI Note 3, H_2_O‐induced rate suppression is about 3–5 times more severe on pure (001) than pure (101), indicating a more pronounced effect on the (001) surface. Since the partial pressure of co‐fed water is orders of magnitude higher than in situ generated water as quantified in Tables S2 and S3, H_2_O inhibition is primarily due to the excessive co‐fed H_2_O but not to the formed H_2_O, which can be further corroborated by the 10%–20% decrease in dehydration rates when co‐feeding only 0.2 kPa water (Figure S5). Figure 2C,D shows dehydration rates as a function of water partial pressure at 250–280°C and a constant IPA pressure of 1 kPa. Dehydration rates decrease with increasing water partial pressure on both facets and level off at roughly 8 kPa. This saturation behavior indicates water cannot displace surface‐adsorbed IPA species but forms alcohol–water complexes that inhibit dehydration. At sufficiently high water and IPA partial pressures, these complexes become the most abundant surface intermediates that exhibit zero orders with respect to IPA and water (Figure 2A,B). From the extent of rate decrease, we again observe stronger water inhibition on TiO_2_(001). Based on these kinetic data, we carried out Arrhenius analysis detailed in Figure S6; the resultant apparent activation barriers *E*_a,j_ (subscript *j* = *001* or *101*, representing TiO_2_(001) or TiO_2_(101)) as a function of water pressure are depicted in Figure 2E,F. On TiO_2_(001), *E*_a,001_ increases from 142 ± 4 kJ mol^−1^ in the absence of co‐fed H_2_O, to 182 ± 5 kJ mol^−1^ at 12 kPa H_2_O. While on TiO_2_(101), *E*_a,101_ increases from 135 ± 1 kJ mol^−1^ (0 kPa H_2_O) to 160 ± 2 kJ mol^−1^ (12 kPa H_2_O). The significantly larger increase in the apparent activation barriers on TiO_2_(001) (40 kJ mol^−1^) compared to TiO_2_(101) (25 kJ mol^−1^) quantitatively confirms the stronger water inhibition on the (001) facet. However, the rates decreased only 5.6 and 2.8‐fold on TiO_2_(001) and (101), respectively. Such modest reductions in rate, despite large enthalpic penalties, imply compensating increases in activation entropy. By using Arrhenius‐based rate ratio expression​ and Eyring equation, the increases in activation entropy are 60 J mol^−1^ K^−1^ for TiO_2_(001) and 35 J mol^−1^ K^−1^ for (101), detailed in Supporting Information: Note 4. The positive change in activation entropy indicates that the presence of water alters the nature of the transition state, increasing its configurational freedom relative to the dry condition, consistent with prior reports [44, 45, 46]. The larger entropy increase observed on TiO_2_(001) compared to TiO_2_(101) suggests that water perturbs the reactive surface intermediate more strongly on the (001) facet. However, the substantial elevation in activation enthalpy due to the dominant kinetic consequence of water leads overall to stronger net rate suppression.

**FIGURE 3:**
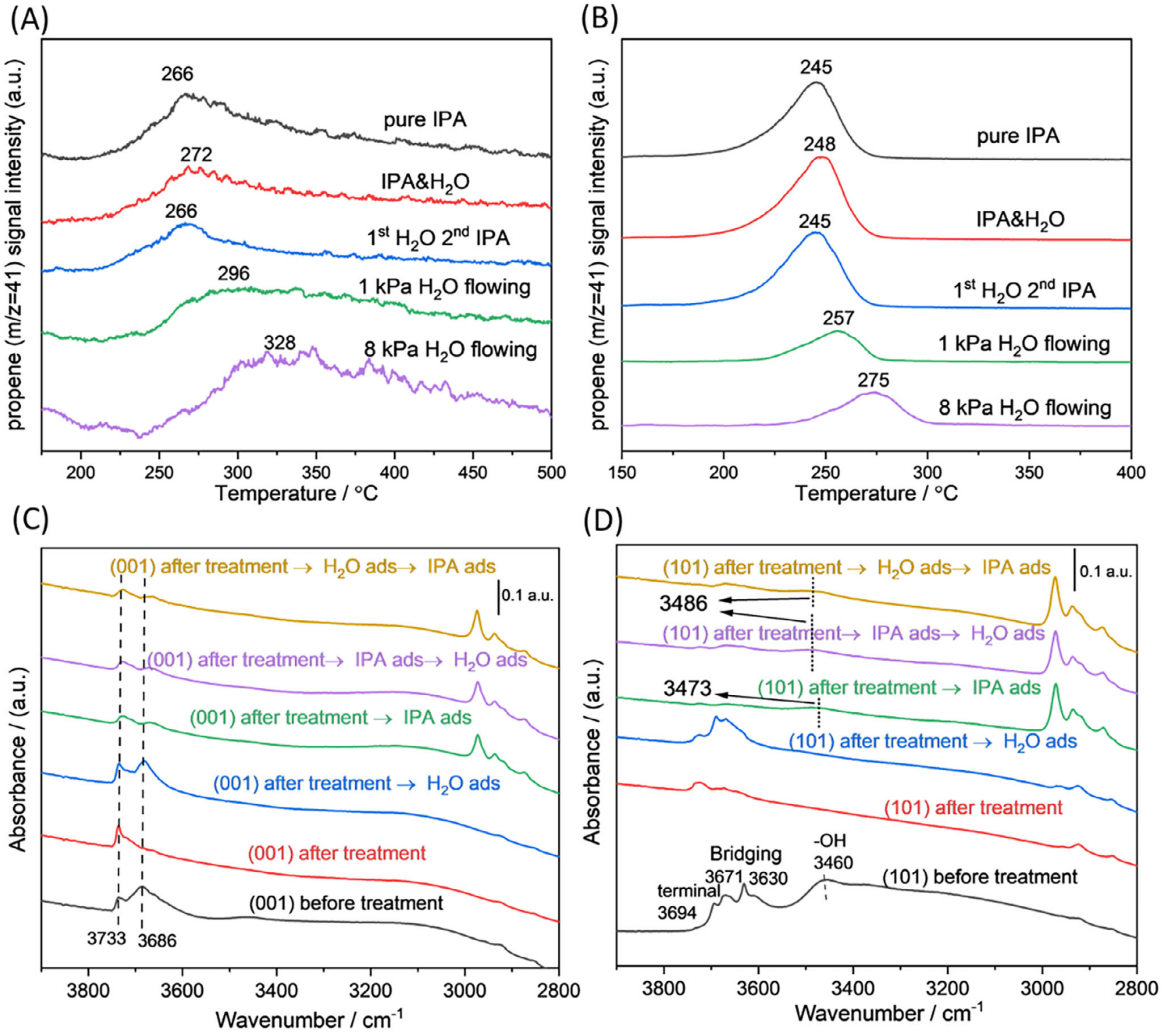
IPA‐TPSR with different ways of introducing water on (A) TiO_2_(001) and (B) TiO_2_(101). High vacuum IR by introducing IPA and H_2_O with different sequences on (C) TiO_2_(001) and (D) TiO_2_(101). Operating conditions: (A), (B) TPSR of pretreated catalysts (450°C, 10% O_2_/He, 1 h) was conducted in flowing He (25 mL min^−1^) with 0 (black, red, blue), 1 (green) or 8 kPa (purple) H_2_O after adsorption of IPA, H_2_O, or H_2_O:IPA mixture (3:2 partial pressure ratio) at 120°C; (C), (D) Pretreated catalysts (red; 450°C, 10^−6^ mbar, 1 h) were initially exposed to IPA (green; 0.1 mbar, 0.5 h), H_2_O (blue; 0.1 mbar, 0.5 h), H_2_O‐to‐IPA or IPA‐to‐H_2_O [yellow or purple; exposure to 0.1 mbar H_2_O (or IPA) for 0.5 h, then degassing at 10^−6^ mbar for 1 h, followed by exposure to 0.1 mbar IPA (or H_2_O) for 0.5 h] at 100°C, followed by vacuum degassing at 10^−6^ mbar for 1 h before transmission IR measurements. Untreated catalysts (black) were degassed at 10^−6^ mbar for 1 h at 25°C.

Figure 3 presents temperature‐programmed surface reaction (TPSR) results using various water introduction methods to probe the facet‐dependent inhibition of IPA dehydration. On TiO_2_(001) (Figure 3A), propene desorption temperature remains unchanged between IPA‐only adsorption and sequential H_2_O‐IPA adsorption (blue trace), indicating that IPA displaces adsorbed H_2_O. However, co‐adsorption of IPA and H_2_O leads to a ∼6°C increase of propene desorption temperature, reflecting water inhibition. Furthermore, introducing H_2_O (1 or 8 kPa) during temperature ramping leads to significant desorption temperature increasing from ∼266 °C to 296 °C or 328 °C, signaling stronger inhibition. Such temperature shifts are indicative of an increased apparent activation barrier [47], consistent with steady‐state kinetic data shown in Figure 2. TiO_2_(101) (Figure 3B) shows similar trends: minimal changes in desorption temperature with different IPA/H_2_O adsorption sequences (black and blue curves) but substantial increases when H_2_O is present during temperature ramping. Notably, the inhibition effect is less pronounced compared to TiO_2_(001), with propene desorption temperatures rising only from ∼245 °C to 257 °C and 275 °C at 1 and 8 kPa H_2_O, respectively. This difference corroborates the larger water‐induced activation barrier increases on TiO_2_(001) compared to TiO_2_(101).

The steady‐state and transient IPA dehydration kinetic results shown above clearly demonstrate more severe H_2_O inhibition on TiO_2_(001). Next, we performed high‐vacuum IR to further exclude the contribution from competitive adsorption between IPA and H_2_O. Four adsorption protocols were employed: (1) H_2_O alone, (2) H_2_O followed by IPA, (3) IPA alone, and (4) IPA followed by H_2_O. On TiO_2_(001) (Figure 3C), preheating at 400°C (red trace) results in a pronounced loss of the hydroxyls compared to the untreated one (black trace), indicating surface dehydroxylation. Exposure of the dehydrated surface to H_2_O (blue trace) restores the bridging hydroxyls. Subsequent IPA dosing to the hydrated surface (yellow trace) produces intense 𝜈(CH) bands (3000–2800 cm^−1^) and a reduction in hydroxyls intensity. This is because at the initial transient state, IPA can react with surface hydroxyl groups, forming surface isopropoxide species and releasing water into the gas phase (Ti(OH) + C_3_H_7_OH = Ti(*i*‐OC_3_H_7_) +H_2_O) [31]. It is worth noting that the coverage of hydroxyl groups becomes dynamically constant after reaching the steady state during the reaction. When IPA is dosed first onto the preheated surface (green trace), similar 𝜈(CH) intensities and hydroxyl consumption are observed. Subsequent H_2_O dosing (purple trace) does not diminish the 𝜈(CH) intensities, demonstrating that adsorbed IPA is not displaced by water. On TiO_2_(101), the dehydrated surface (red trace) likewise exhibits diminished hydroxyl intensity compared to the untreated one (black trace). H_2_O dosing (blue trace) restores bridging hydroxyls. Subsequent IPA dosing (yellow trace) produces strong 𝜈(CH) bands and reduces hydroxyl intensity, with spectral features comparable to when IPA is dosed first (green trace). After adsorbing IPA, subsequent H_2_O exposure (purple trace) does not attenuate the 𝜈(CH) bands, indicating that IPA remains adsorbed. Even a tenfold higher H_2_O pressure does not reduce IPA coverage (Figure S7). Notably, a red‐shifted hydroxyl band at ∼3480 cm^−^^1^ emerges upon IPA adsorption, indicating molecular IPA hydrogen‐bonding with hydroxyls [31]. Upon co‐adsorption of H_2_O either before or after IPA, this hydroxyl feature blue‐shifts from ∼3473 to 3486 cm^−^^1^, demonstrating that water perturbs the IPA–surface hydrogen bonding environment and interacts with co‐adsorbed IPA, forming complexes. These results demonstrate that IPA exhibits stronger adsorption than H_2_O on both TiO_2_ facets. The observed 𝜈(CH) intensities are unaffected by dosing sequence, confirming that competitive adsorption is excluded for contributing to the dehydration inhibition.

### 2.3 Probing IPA‐H_2_O Interactions via In Situ DRIFTS and NMR Spectroscopies

**FIGURE 4:**
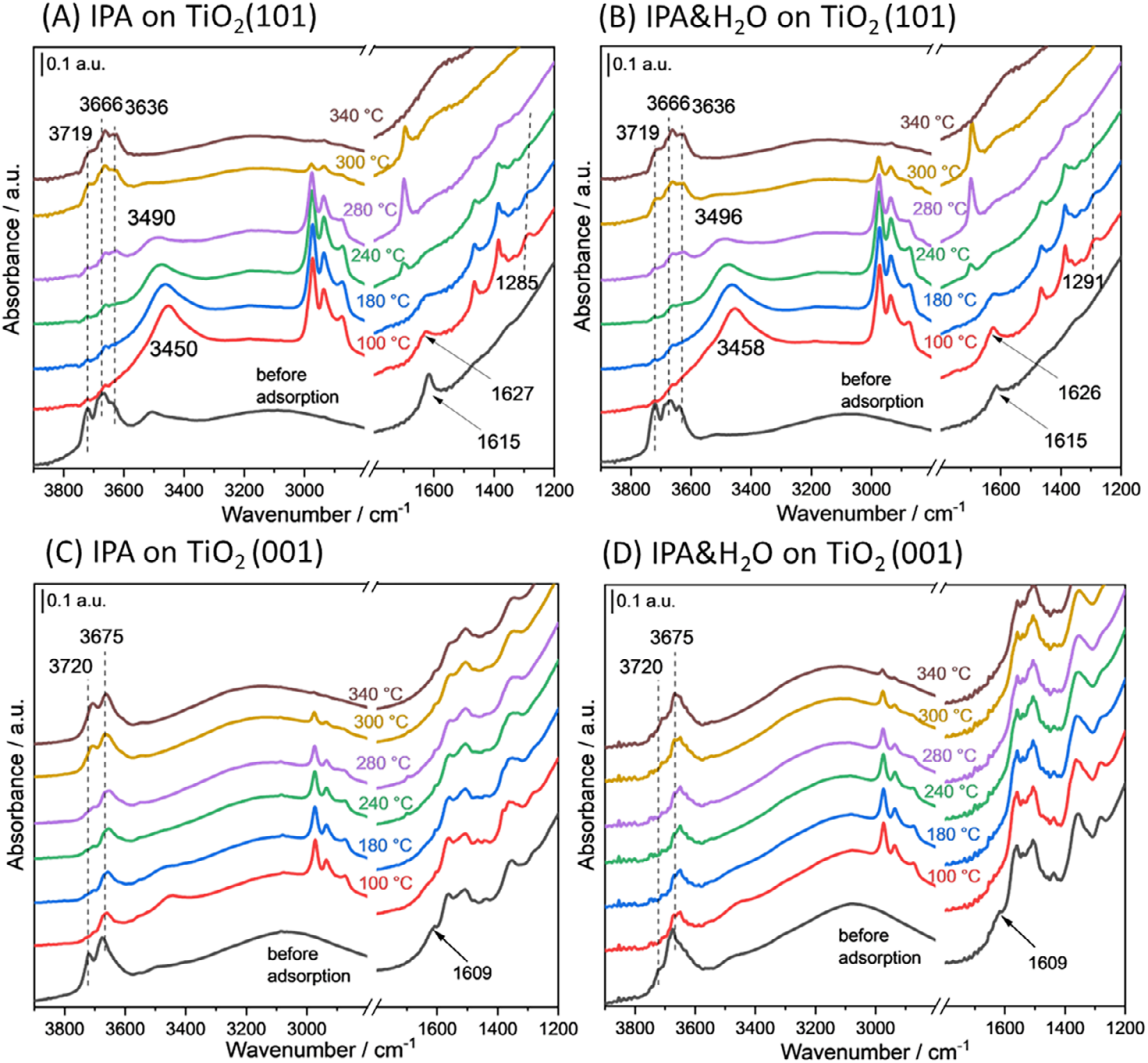
(A) and (C) In situ DRIFTS‐TPD spectra of adsorption and desorption of pure IPA on TiO_2_(101) and (001); (B) and (D) in situ DRIFTS‐TPD spectra of adsorption and desorption of 3:2 H_2_O:IPA (partial pressure) on (101) and (001). Operating conditions: catalysts (20 mg) were pretreated in 10% O_2_/He at 450°C under ambient pressure for 1 h, and then treated with IPA (A, C), or IPA and H_2_O mixture (2:3 partial pressure ratio) (B, D) in flowing He at 100°C until saturation, followed by ramping from 100°C to 450°C (10°C min^−1^) in flowing He (50 mL min^−1^).

Having ruled out competitive adsorption, we employed in situ DRIFTS to investigate the effect of water on adsorption mode of IPA. As shown in Figure 4A, clean TiO_2_(101) (bottom spectrum) presents terminal (3719 cm^−1^) and bridging (3666 and 3636 cm^−1^) hydroxyls [48, 49]. Weak features at ∼3500 and 1615 cm^−^^1^ correspond to residue‐adsorbed H_2_O. A broad IR feature at about 3100 cm^−1^ is also observed for TiO_2_(101). This significantly red‐shifted OH band is characteristic of water molecules adsorbed via strong hydrogen bonding on oxide surfaces [50]. Upon IPA exposure at 100 °C, the original 𝜈(OH) bands at 3636–3719 cm^−1^ largely disappear, and a new 𝜈(OH) band emerges at 3450 cm^−1^, attributed to molecular IPA adsorbed via hydrogen bonding to surface hydroxyls (i.e., Ti‐OH····OH‐C_3_H_7_‐i), evidence by vacuum IR as well. Molecularly adsorbed IPA on Lewis acid sites (Ti_5c_ sites) is further supported by the in‐plane δ(OH) band at 1285 cm^−1^ [51, 52, 53, 54]. IPA adsorption also produces 𝜈(CH_3_)_as_ and 𝜈(CH_3_)_s_ bands within 3000–2800 cm^−1^, as well as δ(CH_3_)_as_ and δ(CH_3_)_s_ bands at 1465 and 1385 cm^−1^, respectively [51, 52, 53]. These vibrational features alone do not distinguish chemisorbed IPA from isopropoxide [53, 55]. It is noted that owing to the sloping baseline and overlap with other vibrational modes, the absorbance in the 1000–1200 cm^−1^ region provides only limited information about the C–O stretching vibrations. Notably, the H_2_O bending mode weakens and blueshifts to 1627 cm^−1^ upon IPA adsorption, indicating partial displacement of pre‐adsorbed water and interaction between the two adsorbates [48]. As temperature increases from 100 to 300 °C, all the IPA bands progressively diminish, suggesting molecular IPA dehydration on Lewis acid‐base pairs via an E2 mechanism [31]. Figure 4B presents spectra acquired upon the co‐adsorption of IPA and water. The perturbed 𝜈(OH) band and in‐plane δ(OH) band shift from 3450 to 3458 cm^−1^ and from 1285 to 1291 cm^−1^ respectively, indicating that water interacts with adsorbed IPA without altering its molecular adsorption mode. The key difference is that 𝜈(CH) peaks vanish at 340°C higher than 320°C in the dry surface case, as shown by the slower decrease in 𝜈(CH) peak areas in Figure S8A, indicating water inhibits dehydration activity, consistent with kinetic results. For pure water adsorption on the (101) facet (Figure S10), the perturbed 𝜈(OH) band appears at 3467 cm^−^^1^, blue shifting about 10 cm^−1^ compared to co‐adsorption case. The shift demonstrates that surface water interacts with adsorbed IPA.

Figure 4C presents spectra with pure IPA adsorption on TiO_2_(001) at 100°C, followed by heating. The clean TiO_2_(001) displays terminal and bridging hydroxyls at 3720 and 3675 cm^−1^. In contrast to TiO_2_(101), a broad 𝜈(OH) feature centered at ∼3100 cm^−1^ is present together with weak H_2_O bending mode at ∼1609 cm^−1^, indicating more rigid and ice‐like H_2_O on TiO_2_(001) [56]. Upon IPA exposure, hydroxyls are partially consumed, accompanied by the appearance of 𝜈(CH) bands. Unlike TiO_2_(101), however, the characteristics of molecular IPA, perturbed 𝜈(OH) (∼3450 cm^−1^) and δ(OH) (∼1290 cm^−1^) features, are barely detected. This indicates that IPA primarily adsorbs dissociatively as isopropoxide [31]. Upon heating, isopropoxide species are fully dehydrated by ∼340°C, accompanied by regeneration of surface hydroxyls. Figure 4D presents spectra with co‐adsorption of IPA and H_2_O. Even at 340°C, characteristic isopropoxide bands remain detectable, indicating water retards isopropoxide dehydration. This inhibitory effect is further quantified by plotting 𝜈(CH) peak area versus temperature (Figure S8B). Importantly, no molecular IPA features emerge under co‐adsorption conditions, indicating that water suppresses isopropoxide dehydration without altering its dissociative adsorption mode. The 1699 cm^−1^ acetone feature is consistent with the trace acetone formed under steady‐state reaction conditions; however, this minor byproduct does not affect our mechanistic conclusions regarding the dominant adsorbed species on the two facets.

**FIGURE 5:**
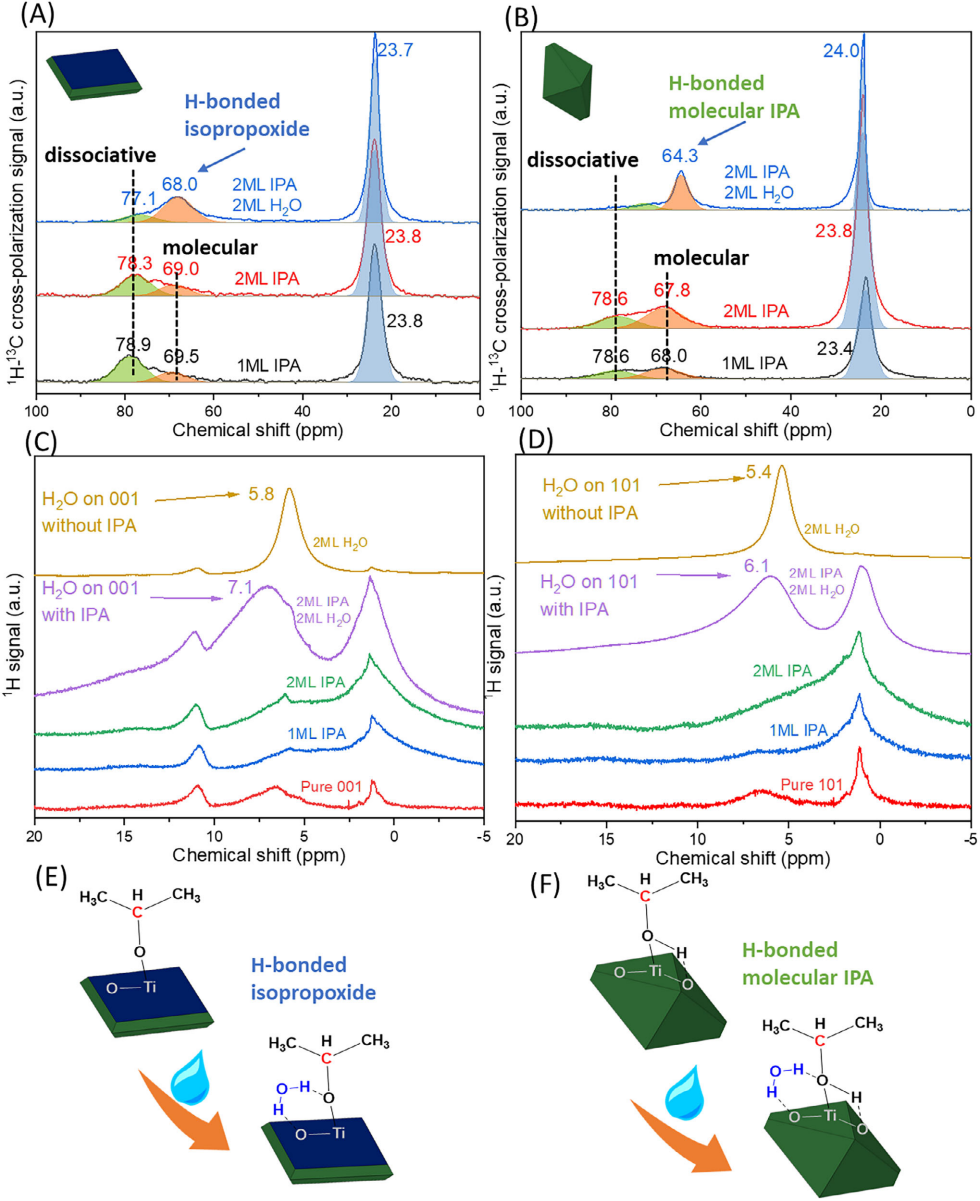
(A) and (B) in situ ^1^H‐^13^C CP NMR of IPA and water adsorbed on (001) and (101); (C) and (D) in situ ^1^H DP NMR of IPA and water adsorbed on TiO_2_(001) and (101); (E) and (F) representative scheme of isopropoxide‐H_2_O complex on (001) and IPA‐H_2_O complex on (101). Operating conditions: catalysts (100 mg) were pretreated in 10% O_2_/He at 450°C under ambient pressure for 1 h. The required amount of IPA and H_2_O liquids were injected onto the samples inside NMR rotor in the glovebox via µL syringes.

While in situ DRIFTS provides valuable insight into facet‐dependent adsorption modes, that is, molecular versus dissociative adsorption, its sensitivity is limited when probing more subtle interactions between IPA and co‐adsorbed water. We further employed in situ ^1^H–^13^C CP and DP MAS NMR spectroscopies, which offer higher sensitivity to the local environment, to probe the molecular‐level interactions among IPA, water, and TiO_2_ surfaces. Figure 5A,B presents ^1^H‐^13^C CP spectra of IPA and water adsorbed on TiO_2_(001) and (101). CP selectively detects strongly adsorbed species while suppressing signals from weakly bound or gas‐phase molecules, a feature evident in Figure 5A, where spectra obtained after dosing TiO_2_(001) with one or two monolayers (ML) of IPA appear nearly identical. A monolayer was defined as the number of surface‐exposed Ti sites quantified by NH_3_‐TPD. Upon IPA adsorption, three CP resonances emerge at ∼78, ∼69, and ∼24 ppm, corresponding to methine carbons of isopropoxide and molecular IPA and methyl groups of both, respectively [57], indicating that NMR allows direct differentiation between molecular IPA and isopropoxide. Deconvolution reveals that ∼80% of adsorbed IPA adopts dissociative binding as isopropoxide, while ∼20% remains molecular. With the addition of an equal amount of water, the intensity of the 78 ppm resonance markedly decreases and that of the 68 ppm peak increases while the total area of the two resonances is largely maintained. Since high‐vacuum IR demonstrates no competitive adsorption, the 68 ppm resonance observed in the presence of 2 ML H_2_O is unlikely to arise from molecular IPA but is more plausibly an isopropoxide–H_2_O complex, as depicted in Figure 5E. Additionally, ∼24 ppm methyl resonance became much sharper, indicating the faster molecular motion of the methyl group. Methine ^13^C of IPA upshifting and a sharper methyl ^13^C peak both indicate weaker surface binding of isopropoxide and a more disordered adsorbate environment with the presence of water, leading to a possibly loosened and disordered configuration.

In contrast, upon dosing TiO_2_(101) with IPA (Figure 5B), adsorption is dominated by the molecular state at ∼68 ppm (∼70%), with a minor dissociative isopropoxide contribution of ∼78 ppm (∼30%), consistent with DRIFTS. Upon co‐adsorption with H_2_O, the dominant molecular IPA resonance shifts from ∼68 to ∼64 ppm. Since H_2_O does not compete with IPA on TiO_2_(101) either, this peak is assigned to a hydrogen‐bonded molecular IPA complex. This assignment is corroborated by ^13^C DP spectra at 12 ML IPA loadings (Figure S12C), which show a significantly sharp signal at ∼64 ppm from weakly bound molecular IPA that is absent in ^1^H–^13^C CP, confirming the ∼64 ppm CP resonance corresponds to hydrogen‐bonded molecular IPA. A ∼10 ppm shift on TiO_2_(001) compared to a ∼4 ppm shift on TiO_2_(101) indicates a stronger interaction between isopropoxide‐water on the (001) surface than IPA‐water on the (101) surface. Again, the methine ^13^C of IPA upshifting and a sharper methyl ^13^C peak both indicate that water weakens the adsorption of molecular IPA on the surface and creates a more disordered adsorbate environment. We further conduct DFT‐NMR to cross‐validate the assignment of IPA species adsorbed. As shown in Figure S13, the isopropoxide resonates at 86.4 ppm, which upshifts by 5.3 to 81.1 ppm with the presence of one water molecule, shown in Models (B) and (C). In contrast, molecularly adsorbed IPA shifts from 71.8 by 1.8 to 70.0 ppm with the presence of water. These trends align with experimental observations, confirming that water perturbs isopropoxide more strongly than molecular IPA on two respective facets.

To further support this interpretation, we conduct ^1^H MAS NMR (Figure 5C,D). Clean TiO_2_(001) and (101) display terminal hydroxyls at ∼1 ppm and bridging hydroxyls at ∼6.5 ppm. TiO_2_(001) exhibits an additional resonance at ∼11 ppm, which has been assigned to bicarbonate species [58]. These species may originate from interactions between hydroxyl groups and CO_2_ generated from urea decomposition during hydrothermal synthesis and subsequent calcination [58, 59]. However, because this resonance remains largely unchanged upon IPA and H_2_O adsorption, it appears that these species interact minimally, if at all, with IPA or water under the conditions studied. Therefore, this feature is unlikely to significantly affect our main conclusions regarding the facet‐dependent influence of distinct IPA–H_2_O complexes on reaction kinetics. Upon IPA adsorption, the ∼1 ppm signal increases and broadens relating to methyl groups of IPA, while the ∼6.5 ppm resonance weakens and becomes less defined due to interacting with IPA through hydrogen bonding or forming isopropoxide, as illustrated in ^13^C NMR and DRIFTS. Following the adsorption of 2 ML of H_2_O, water proton resonances appear at 7.1 ppm on TiO_2_(001) and 6.1 ppm on TiO_2_(101). In contrast, H_2_O adsorbed alone on the clean surfaces yields resonances at 5.8 and 5.4 ppm, respectively. The larger downfield shift observed on TiO_2_(001) (Δ = 1.3 ppm) compared to TiO_2_(101) (Δ = 0.7 ppm) indicates a stronger deshielding effect by neighboring IPA/isopropoxide, indicating stronger hydrogen bonding between H_2_O and the oxygen of isopropoxide on the (001) facet. In comparison, the smaller shift on TiO_2_(101) suggests weaker hydrogen bonding between H_2_O and molecular IPA. Combining ^1^H‐^13^C CP and ^13^C and ^1^H DP NMR, we propose that water interacts with isopropoxide on TiO_2_(001) to form an isopropoxide‐H_2_O complex intermediate (Figure 5E). In contrast, H_2_O interacts weakly with molecular IPA on (101) to form hydrogen‐bonded molecular IPA intermediate (Figure 5F). Next, we apply DFT calculations and kinetic analysis to support such structure models and discuss how this molecular‐level knowledge helps to better understand H_2_O inhibition in acid‐base catalysis.

### 2.4 Mechanistic Considerations of Facet‐Dependent Inhibition From DFT Calculations and Kinetic Studies

Previous studies have established that IPA dehydration on TiO_2_ prefers to undergo via an E2 elimination mechanism, where the rate‐limiting step is concurrent C‐H and C‐O bond cleavages [31, 60]. To reveal potential changes of reaction mechanism under H_2_O inhibition, we carried out kinetic isotope effect (KIE) measurements using CD_3_CH(OH)CD_3_ or CH_3_CH(OD)CH_3_ at 260°C. As demonstrated in Table S4, when hydroxyl hydrogen is deuterated, KIEs are approximately unity on both TiO_2_(001) and (101), regardless of the presence or absence of co‐fed water, indicating no isotope effect. However, when C_β_‐H are deuterated, KIEs are close to 2 on both TiO_2_(101) and (001) with and without co‐fed water. These results suggest that the reaction in the presence of water still follows the E2 mechanism.

**FIGURE 6:**
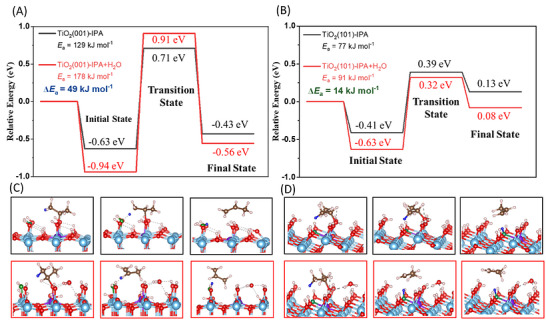
DFT calculations of reaction pathway and activation energy of IPA dehydration with and without water on (A) TiO_2_(001) and (B) TiO_2_(101). Initial, transition, and final states models with and without water on (C) TiO_2_(001) and (D) TiO_2_(101).

Besides, since in situ DRIFTS and NMR spectroscopies consistently demonstrate that IPA adsorbs predominantly molecularly on TiO_2_(101) and dissociatively on TiO_2_(001) with water addition not altering these adsorption modes, DFT calculations were performed to corroborate these assignments and to rationalize the facet‐dependent water inhibition mechanisms observed experimentally. According to the previous study [31], Ti‐O_s_‐Ti(OH) and Ti‐O_s_ are proposed as active sites on TiO_2_(001) and (101), respectively. As shown in Figure 6A,C, IPA dissociates spontaneously on active sites (Ti‐O_s_‐Ti(OH)) of (001) surface, forming Ti(*i*‐OC_3_H_7_)‐O_s_(H)‐Ti(OH), where the Ti‐O bond distance in the Ti(*i*‐OC_3_H_7_) moiety is 1.8 Å, and the C_β_‐C_α_‐Ti angle is 109°, that is, the carbon chain lies relatively parallel to the surface, as depicted in Figure S14A. The transition state is formed by Ti‐OH····H‐C_β_ interactions with a computed activation barrier of 129 kJ mol^−1^. The simultaneous C‐H and C‐O cleavage of this transition state (i.e., E2 dehydration) yields propene and water. In the presence of an H_2_O molecule, it interacts with Ti(*i*‐OC_3_H_7_) via hydrogen bonding through the O atom of isopropoxide, according to NMR analysis. The H‐O‐H····O‐Ti hydrogen bond distance is 1.84 Å, that is, a relatively strong hydrogen bonding, consistent with ^1^H NMR. It is also worth noting that (1) the Ti‐O bond distance of the intermediate is elongated from 1.80 to 1.88 Å upon water coordination and (2) the C_β_‐C_α_‐Ti angle is enlarged to 122° (Figure S14B), consistent with NMR results that the H_2_O molecule pushes methyl groups away from the (001) surface. Consequently, the methyl groups tilt away from the TiO_2_(001) surface. This geometric distortion reflects the weakened adsorption of the reacting isopropoxide species, which makes the transition state less stabilized by the surface, leading to a markedly higher activation energy of 179 kJ mol^−1^. The activation barrier difference of 50 kJ mol^−1^ resulting from water inhibition is consistent with experimental results (40 kJ mol^−1^) shown in Figure 2E. Although entropy was not explicitly evaluated in the DFT calculations, the predicted geometric relaxation and reduced surface confinement are consistent with the experimentally observed increase in activation entropy. Formation of the isopropoxide–water complex weakens the rigidity of the adsorbed species and likely induces greater configurational flexibility in the transition state. Thus, the DFT results support a mechanistic picture where water destabilizes the transition state with an increasing structural disorder.

Figure 6B,D presents the energy profile of the E2 elimination step and the corresponding models on the (101) surface, respectively. For the molecular adsorbed Ti(IPA)‐O_s_, the Ti‐O distance in the Ti(IPA) moiety is 2.12 Å, obviously longer than isopropoxide on TiO_2_(001). The two C_β_‐C_α_‐Ti bond angles are 94° and 95°, parallel to the (101) surface (Figure S14C). The transition state is stabilized by lattice O_s_ and Ti, derived from previous reported models [31]. The calculated activation energy is 77 kJ/mol, lower than that on the (001) surface, in agreement with kinetic measurements and prior theoretical studies [31]. After introducing an H_2_O molecule, the Ti‐O distance of the Ti(IPA···H_2_O) moiety is elongated to 2.21 Å and two C_β_‐C_α_‐Ti angles expand to 97 and 137°, indicating partial tilting of one methyl group away from the surface (Figure S14D). The hydrogen bond distance of H‐O‐H····O‐Ti in the Ti(IPA···H_2_O) moiety is 2.15 Å, much longer than the hydrogen bond distance (1.84 Å) found on (001) surface. As such, the much weaker water inhibition on TiO_2_(101) only leads to an increase in activation energy of E2 elimination of 14 kJ mol^−1^, which is also in line with experimental data (25 kJ mol^−1^). The geometry of the (101) surface and its preference for molecular IPA adsorption likely limit the perturbance degree of water on the reactive intermediate, thereby reducing its influence on the transition state. Although entropy was not explicitly calculated, the relatively small structural distortion upon water coordination suggests a smaller increase in configurational flexibility of the transition state, consistent with the smaller experimental increase in activation entropy compared to the (001) case.

The combination of kinetic studies, in situ DRIFTS and NMR characterizations, isotope studies, as well as DFT calculations, allows us to elucidate the elementary steps of IPA dehydration on TiO_2_(001) and (101), as depicted in Tables S5 and S6. On TiO_2_(001), the reaction initiates with the dissociative adsorption of IPA, forming isopropoxide (Ti(*i*‐OC_3_H_7_)‐O_s_(H)‐Ti(OH), **Step (001)‐1**). Meanwhile, the molecular adsorption of H_2_O generates Ti(H_2_O)‐O_s_‐Ti(OH) species (**Step (001)‐2**). The adsorbed species can interact with another gaseous H_2_O molecule, forming an isopropoxide‐water complex (Ti(*i*‐OC_3_H_7_···H_2_O)‐O_s_(H)‐Ti(OH), **Step (001)‐3**) or a water dimer (Ti(H_2_O)_2_‐O_s_‐Ti(OH), **Step (001)‐4**). Subsequently, the kinetically relevant E2 elimination occurs via the attack of the OH to the C_β_‐H of the *i*‐OC_3_H_7_ moiety and the simultaneous C‐O bond cleavage, releasing propene into the gas phase (**Steps (001)‐m‐5** and **(001)‐d‐5**, where *m* and *d* denote monomeric and complex pathways, respectively). Finally, active sites are regenerated by the desorption of H_2_O (**Steps (001)‐m‐6** and **(001)‐d‐6**).

On TiO_2_(101), the reaction begins with molecular adsorptions of IPA or H_2_O on Ti‐O_s_, forming Ti(IPA)‐O_s_ (**Step (101)‐1**) and Ti(H_2_O)‐O_s_ species (**Step (101)‐2**), respectively, which can interact with another H_2_O molecule to generate a IPA‐water complex (Ti(IPA···H_2_O)‐O_s_), **Step (101)‐3**) or a water dimer (Ti(H_2_O)_2_‐O_s_), **Step (101)‐4**). Subsequently, Ti(IPA)‐O_s_ and Ti(IPA···H_2_O)‐O_s_ undergo via E2 elimination by the attack of O_s_ to the C_β_‐H of the IPA moiety and by the simultaneous C‐O bond cleavage, producing gaseous propene (**Steps (101)‐m‐5** and **(101)‐d‐5**, respectively). Finally, the desorption of H_2_O regenerates the Ti‐O_s_ active sites to complete the catalytic cycle (**Steps (101)‐m‐6** and **(101)‐d‐6**).

Since the E2 elimination is the kinetically relevant step for both monomeric (**Steps (001)‐m‐5** and **(101)‐m‐5**) and complex pathways (**Steps (001)‐d‐5** and **(101)‐d‐5**), the pseudo steady‐state approximation of all surface species leads to the following rate expression with the detailed derivation provided in Supporting Information: Note 5,

(1) $$\begin{array}{c} r_{j}=r_{m,j}+r_{d,j}=\frac{k_{m,j}K_{I,j}\left[IPA\right]+k_{d,j}K_{W\cdot D,j}K_{I,j}\left[IPA\right]\left[H_{2}O\right]}{1+K_{I,j}\left[IPA\right]+K_{W1,j}\left[H_{2}O\right]+K_{W2,j}{\left[H_{2}O\right]}^{2}+K_{W\cdot D,j}K_{I,j}\left[IPA\right]\left[H_{2}O\right]}\; \end{array}$$

where *k*_m,j_ and *k*_d,j_ are the rate constants for monomer and complex; *K*_I,j_, *K*_w1,j_ *K*_w·D,j_ are the equilibrium constants for **Steps (j)‐1**, **(j)‐2**, and **(j)‐3**, respectively; and *K*_w2,j_ is the overall equilibrium constants for **Steps (j)‐2 and (j)‐4**. Nonlinear regression of the measured data to Equation (1) yields the parameters shown in Table S7, with the parity plots and sensitivity analysis provided in Figures S15–S17. Fitted results presented as dashed lines in Figure 2 match well with experimental data.

On TiO_2_(001), the rate constant of the monomeric pathway (*k*_m,001_​) is 1.56 (± 0.17) × 10^−2^ s^−1^, which is 6.3 times higher than that of the complex pathway (*k*_d,001_), 2.49 (± 0.15) × 10^−3^ s^−1^. Distinctively, on TiO_2_(101), *k*_m,101_ and *k*_d,101_ are 2.87 (± 0.03) × 10^−2^ and 7.10 (± 0.90) × 10^−3^ s^−1^, respectively, with the *k*_m,101_(*k*_d,101_)^−1^ ratio of 4.0. The larger *k*_m,001_(*k*_d,001_)^−1^ than *k*_m,101_(*k*_d,101_)^−1^ ratio agrees with the larger activation barrier difference on TiO_2_(001) than on TiO_2_(101). The *k*_m,101_(*k*_m,001_) ratio of 1.8 indicates that the monomeric pathway is easier to occur on TiO_2_(101) than on TiO_2_(001), agreeing with the lower activation barrier shown in Figure 2E,F, since molecularly adsorbed IPA on (101) is more favorable to dehydration than dissociatively adsorbed IPA on (001) [31]. Besides, the much larger *K*_w·D,j_ simulated on TiO_2_(001) than on TiO_2_(101) is consistent with the stronger adsorption energy of the isopropoxide‐water complex on the former than that of the IPA‐water complex on the latter. As shown in Table S8, for the coverage of the complex at 2–8 kPa H_2_O, although it is similar on both facets at low IPA pressures (0.25‐1 kPa), it is higher on TiO_2_(001) (72%‐91%) than on TiO_2_(101) (54%–82%) at higher IPA pressures (2‐8 kPa IPA). Besides, the complex pathway becomes dominant (r_d,j_(r_m,j_)^−1^ > 1) at above 2.3 kPa and 5.8 kPa H_2_O on TiO_2_(001) and (101), respectively, indicating that TiO_2_(001) is more sensitive to the co‐fed water. Collectively, the stronger water inhibition on IPA dehydration rates on TiO_2_(001) arises from both a greater suppression of the rate constant of the complex pathway and the dominant surface coverage of isopropoxide‐water species in a wide range of operating conditions.

## 3 Conclusion

Unraveling how water influences alkanol dehydration is critical for advancing biomass upgrading under realistic and water‐rich conditions. By exploiting TiO_2_ facet engineering to minimize surface heterogeneity, this work provides an unambiguous molecular‐level picture of how water inhibits IPA dehydration in a facet‐dependent manner. We demonstrate that water suppresses the IPA dehydration rate on TiO_2_(001) surface much more severely than on TiO_2_(101), revealing fundamentally distinct inhibition mechanisms. Spectroscopic and kinetic analyses rule out competitive adsorption as an inhibition contributor. Instead, combined in situ IR, in situ NMR, kinetic studies, and DFT calculations reveal that water interacts strongly with dissociatively adsorbed isopropoxide on TiO_2_(001), forming an isopropoxide–H_2_O complex that readily drives the surface into a complex‐dominated regime and generates a more disordered and loosely bound transition state, which can greatly elevate the barrier by 40 kJ mol^−1^. In contrast, on TiO_2_(101), water interacts only weakly with molecularly adsorbed IPA through hydrogen bonding with much lower coverages, leading to a smaller complex formation constant and a much smaller increase in the activation barrier (25 kJ mol^−1^). This work identifies facet‐dependent solvation of surface intermediates as a control parameter, demonstrating that water can fundamentally reshape activation barriers in a facet‐specific manner and offering a new conceptual basis for catalyst design under realistic and water‐rich conditions.

## Author Contributions

**Wenda Hu**: writing – original draft, investigation, methodology, writing – review and editing. **Haiting Cai**: writing – original draft, formal analysis, data curation, writing – review and editing. **Anthony Savoy**: investigation, methodology, software. **Jinshu Tian**: data curation, formal analysis, visualization. **Sungmin Kim**: investigation, resources. **Fan Lin**: methodology, investigation. **Junrui Li**: methodology, validation. **Hao Xu**: formal analysis. **Yiqing Wu**: methodology. **Zihao Zhang**: writing – review and editing. **Nicholas Jaegers**: methodology, writing – review and editing, resources, software. **Feng Gao**: supervision, writing – review and editing, resources. **Huamin Wang**: resources, conceptualization, supervision. **Jianzhi Hu**: supervision, writing – review and editing, resources, funding acquisition, conceptualization. **Yong Wang**: supervision, writing – review and editing, resources, funding acquisition, conceptualization, project administration.

## Conflicts of Interest

The authors declare no conflicts of interest.

## Supporting information



The authors have cited additional references within the Supporting Information.**Supporting File 1**: anie73033‐supp‐0001‐SuppMat.docx.

## Data Availability

The data that support the findings of this study are available from the corresponding author upon reasonable request.
