# Supplementary material for: Facet‐Dependent Water Inhibition of Alkanol Dehydration on TiO2 via Distinct Water–Alkanol Complexes
Source: Angew Chem Int Ed Engl. 2026 Jun 7;65(29):e2431054. doi: 10.1002/anie.2431054 (PMC13360757; doi:10.1002/anie.2431054)
Supplement: Supplementary file 1 — The authors have cited additional references within the Supporting Information.Supporting File 1: anie73033‐supp‐0001‐SuppMat.docx. [file ANIE-65-e2431054-s001.docx]

**Facet-Dependent Water Inhibition of Alkanol Dehydration on TiO_2_ via Distinct Water–Alkanol Complexes**

**Supporting information**

**Table of contents**

1. **Experimental section**
2. **Notes**

Note 1. Mass Transfer Calculation
Evaluation of internal and external diffusion limitations (Weisz–Prater and Mears criteria).

Note 2. Facet Analysis
Quantitative (001)/(101) surface fraction estimation by TEM image measurements.

Note 3. Extrapolation of Facet-Dependent Inhibition
Derivation and worked example of inhibition ratio as a function of (001) surface fraction.

Note 4. Activation Entropy Compensation Calculations
Entropy–enthalpy compensation analysis using transition-state theory (Eyring equation).

Note 5. Derivation of Turnover Rate Expression
Detailed kinetic model including IPA monomer, IPA-H_2_O complex and site balance derivation.

1. **Figures**

Figure S1. XRD patterns of fresh TiO_2_(001) and (101) samples

Figure S2. TEM images of (A) (001) with nanosheet shapes and (B) (101) with bipyramidal shapes with facet ratio measurement analysis. (C) and (D) TEM images of (001) and (101) after reaction demonstrating no structural change and remained facet ratios.

Figure S3. (A) NH_3_-TPD and (B) CO_2_-TPD profiles of TiO_2_(101) and (001).

Figure S4. Extrapolation of inhibition ratios vs. (001) fraction at different water pressures (2, 4, 8 kPa).

Figure S5. Kinetic dependence of IPA dehydration on TiO_2_(001) (A) in the absence of water and (B) in the presence of water (0.2 kPa water). Operating conditions: 240-270 ^o^C,1 atm total pressure with 0.1-7 kPa IPA, 0.2 kPa co-fed H_2_O, and balance He, and 47.6 cm^3^ g^-1^ s^-1^ GHSV.

Figure S6. Arrhenius plots of IPA dehydration on (A) TiO_2_(001) and (B) (101) at 1 atm total pressure with 1 kPa IPA, 0-12 kPa co-fed H_2_O and 47.6 cm^3^ g^-1^ s^-1^ GHSV.

Figure S7. High vacuum IR by introducing IPA and H_2_O with increasing pressure from 0.1 mbar to 1.1 mbar on (101).

Figure S8. C-H vibration peak area integrated from DRIFTS spectra versus temperature with pure IPA adsorption and H_2_O: IPA partial pressure ratio of 3:2 on (A) (101) and (B) (001)

Figure S9. DRIFTS spectra of -OH region with (bottom) pure IPA on (101) and (top) H_2_O&IPA on (101).

Figure S10. (A) and (B) in situ DRIFTS-TPD spectra of adsorption and desorption of pure H_2_O on (101) and (001)

Figure S11. Thermodynamic equilibria calculations of IPA dehydration at temperature range from 250-280 °C with or without co-fed water (water:IPA = 4:1 or 0:1, respectively)

Figure S12. *In situ* ^13^C direct polarization NMR of IPA and water adsorbed on (A) (001) and (B) (101); (C)^13^C NMR of different coverages IPA on (001) at 25 °C(DP) and 110 °C(CP)

Figure S13. (A) DFT-NMR simulated ^13^C NMR spectra of (B) isopropoxide, (C) isopropoxide + H_2_O, (D) isopropanol, and (E) isopropanol + H_2_O adsorbed on TiO_2_.

Figure S14. Zoom in model structures illustrating bond angles and bond distances with (A) IPA on TiO_2_ (001), (B) IPA+H_2_O on TiO_2_ (001), (C) IPA on TiO_2_ (101) and (D) IPA+H_2_O on TiO_2_ (101).

Figure S15. Parity plots for the predicted (from eq. S33 and parameters in Table S7) and measured turnover frequencies on (A) TiO_2_(101) and (B) (001) at 260 °C, 1 atm total pressure with 0.25-8 kPa IPA, 0-8 kPa H_2_O, balance He, and 47.6 cm^3^ g^-1^ s^-1^ GHSV. The solid line represents a perfect prediction of turnover frequencies for propene formation

Figure S16. Sensitivity of sum of squares of residuals to variations in optimal kinetic and thermodynamic parameters of the rate expression (Table S3, eq. S33) on TiO_2_ (001) at 260 ^o^C,1 atm total pressure with 0.25-8 kPa IPA, 0-8 kPa H_2_O, and balance He, and 47.6 cm^3^ g^-1^ s^-1^ GHSV.

Figure S17. Sensitivity of sum of squares of residuals to variations in optimal kinetic and thermodynamic parameters of the rate expression (Table S3. eq. S33) on TiO_2_ (101) at 260 ^o^C,1 atm total pressure with 0.25-8 kPa IPA, 0-8 kPa H_2_O, and balance He, and 47.6 cm^3^ g^-1^ s^-1^ GHSV.

1. **Tables**

Table S1. Length measurement and (001) percentage calculations

Table S2. Turnover frequencies and conversions of IPA dehydration on TiO_2_(001) at 260 °C, 1 atm total pressure with 0.25-8 kPa IPA, 0-8 kPa H_2_O, balance He, and 47.6 cm^3^ g^-1^ s^-1^ GHSV.

Table S3. Turnover frequencies and conversions of IPA dehydration on TiO_2_(101) at 260 °C, 1 atm total pressure with 0.25-8 kPa IPA, 0-8 kPa H_2_O, balance He, and 47.6 cm^3^ g^-1^ s^-1^ GHSV.

Table S4. Kinetic isotope effect of IPA dehydration using CD_3_CH(OH)CD_3_ or CH_3_CH(OD)CH_3_ with co-fed water on TiO_2_(001) and (101) at 260 °C. 1 atm total pressure with 1 kPa reactant, 4 kPa H_2_O, and balance He, and 47.6 cm^3^ g^-1^ s^-1^ gas hourly space velocity (GHSV).

Table S5. Elementary Steps of IPA dehydration on TiO_2_(001)

Table S6. Elementary Steps of IPA dehydration on TiO_2_(101)

Table S7. Kinetic and thermodynamic parameters obtained by the non-linear fitting of experimental data to rate expressions (eq. S33). Operating conditions: 260 °C, 1 atm total pressure with 0.25-8 kPa IPA, 0-8 kPa H_2_O, balance He, and 47.6 cm^3^ g^-1^ s^-1^ GHSV.

Table S8A. The fractional coverage of surface species and turnover frequencies of monomeric and complex pathway on TiO_2_(001) at 260 °C (calculated from eq. S33 and parameters in Table S7).

Table S8B. The fractional coverages of surface species and turnover frequencies of monomeric and complex pathway on TiO_2_(101) at 260 °C (calculated from eq. S33 and parameters in Table S7).

1. **Experimental section**

**Catalysts preparation**

TiO_2_(101) and (001) nanocrystals with well-defined morphology and respective dominant (101) and (001) facet exposure were hydrothermally synthesized via a modified two-step approach.^[62-64]^ First, potassium titanate precursor was synthesized as follows: 22.4 g of KOH (Sigma-Aldrich, ≥ 85 wt %) was dissolved in 40 mL of deionized water, followed by adding 1 g of P25 (Aldrich, nanopowder, 21 nm in diameter, ≥ 99.5 wt %) while stirring. The obtained suspension was transferred into a Teflon-lined stainless-steel autoclave which was kept at 200 °C for 24 h. After synthesis, the white precipitate was treated by repeated washing-centrifugation five times and then dried at 80 °C overnight. TiO_2_(101) was prepared by adding 0.2 g of the precursor to 250 mL of deionized water and sonicating for 45 min. The obtained solution was sealed in a Teflon-lined stainless-steel autoclave, and hydrothermal synthesis was conducted at 200 °C for 24 h. For the synthesis of TiO_2_(001), after sonicating the same precursor/H_2_O suspension for 45 min, 45 g of urea (Sigma-Aldrich, ACS reagent) was added as a capping agent. In this case, hydrothermal synthesis was carried out at 200 °C for 20 h. The as-synthesized catalysts were washed by 1.6 M HNO_3_ followed by centrifugation separation two times to remove residual potassium, and then with repeated washing-centrifugation using deionized water for five times, dried in air at 80 °C overnight and calcined in the muffle furnace at 550 °C for 4 h.

**Catalyst characterizations**

The X-ray diffraction (XRD) analyses for the fresh TiO_2_(001) and (101) catalysts were performed using a Rigaku Miniflex-II diffractometer (CuKα radiation) over a 2θ range of 10°-80°, with a scan speed of 2° min^-1^, a voltage of 30 kV and a current of 15 mA.

Surface area measurements were conducted on a QuantaChrome Autosorb-6 using N_2_ adsorption isotherms and BET analysis methods. Samples were degassed in vacuum at 150 °C for 4 h before adsorption measurements. TEM was carried out using a JEOL JEM 2010 system operated at 200 keV. The TEM specimens were prepared by dispersing calcined TiO_2_ samples in ethanol and depositing the suspension onto a lacey carbon-coated copper grid.

Temperature-programmed desorption (TPD) of various probe molecules was performed using a chemisorption unit (AutoChem II 2920 Chemisorption Analyzer, Micromeritics) coupled with an online QMS (Quadra 220, Pfeiffer). Each sample was first pretreated at 450 °C under He flow (50 mL min^-1^) for 1 h before cooling to 100 °C and exposing the sample to the probe molecule. Ammonia-TPD (NH_3_-TPD) was conducted by exposing the sample to 1% NH_3_/He (50 mL min^-1^) until saturation. The sample was purged under He flow (50 mL min^-1^) for 2 h to remove physically adsorbed ammonia. The temperature then increased to 700 °C at a ramping rate of 10 °C min^-1^ under He flow (50 mL min^-1^). The quadrupole mass spectrometer was used to measure the desorbed NH_3_ (m/z = 16, 17) during TPD.

Transmission infrared (IR) spectra of TiO_2_(101) and TiO_2_(001) catalysts were recorded using a Nicolet iS50 FT-IR spectrometer (Thermo Fisher Scientific) at a resolution of 4 cm^-1^. The catalyst powders were pressed into self-supporting wafers and placed in a custom-designed *in situ* IR cell, allowing the infrared beam to pass through the center of the wafer. FTIR spectra are presented in absorbance units (log(1/T), where T denotes transmittance). Identical catalyst mass, pellet preparation procedure, and optical conditions were used for all measurements. Spectra were baseline-corrected, and comparisons are therefore made under consistent experimental conditions. Scale bars were provided in the Figures. Prior to spectral acquisition, the samples were thermally activated under high vacuum (10*^-6^* mbar) at 450 °C (10 °C/min of ramping rate) and held for 1 h. After cooling to 100 °C under high vacuum, baseline spectra were acquired against a blank background. Adsorption studies with water and IPA were performed using two distinct sequential protocols: for sequence (i) of water-to-IPA, the activated catalyst was exposed to 0.1 mbar of water vapor for 0.5 h at 100 °C, followed by vacuum outgassing (10*^-6^* mbar) for 1 h, and subsequently exposed to 0.1 mbar IPA vapor for 0.5 h and outgassed again for 1 h; and for sequence (ii) of IPA-to-water, the activated catalyst was first exposed to 0.1 mbar IPA vapor for 0.5 h at 100 °C, followed by outgassing for 1 h, and then subjected to 0.1 mbar water vapor for 0.5 h, followed by another outgassing step. In both protocols, spectra were collected after vacuum outgassing to remove physisorbed species, ensuring the measurement of chemisorbed or strongly bound molecules. All transmission measurements were performed at 100 °C using the same blank background to ensure consistency across all experiments. These conditions allowed for detailed evaluation of the adsorption ability of IPA and water on TiO_2_ surfaces.

*In situ* DRIFTS spectra of the IPA:H_2_O mixtures were conducted using a Bruker Tensor 27 FTIR spectrometer equipped with an *in situ* cell (Harrick Scientific Products inc.). Approximately 20 mg of sample in the fine powder form was loaded into the sample holder and pretreated in 20 mL/min 10% O_2_/He at 450 °C for 1 h to remove adsorbed water and other impurities. Sample was then progressively cooled to 100 °C at 10 °C min^-1^ during which, background spectra were acquired at several designated temperatures by pausing the sample cooling. A complementary set of background scans were also acquired using KBr and TiO_2_ catalysts. Solutions of IPA:H_2_O were introduced by flowing 10 mL min^-1^ He through a bubble generator until saturation (i.e., spectra become invariant with time). The cell was then purged with 50 mL min^-1^ He to remove physiosorbed species from the surface. Sample temperature was then ramped to 450 °C at 10 °C min^-1^, during which spectra were acquired at designated temperatures. Each spectrum was analyzed using the clean sample or KBr backgrounds.

*In situ* Magic Angle Spinning (MAS) ^1^H and ^13^C NMR measurements were conducted on a Varian-Agilent Inova wide-bore 300 MHz NMR spectrometer using a commercial 7.5 mm Vespel pencil type MAS probe, operated at ^1^H and ^13^C Larmor frequencies of 299.93 and 75.42 MHz, respectively.^46^ A 7.5 mm home-made all zirconia *in situ* MAS NMR rotor^[65-68]^ was used to house mixtures of TiO_2_ powder, IPA, and water. The faceted TiO_2_ samples were calcined in 10% O_2_/He (50 mL min^-1^) at 450 °C for 1 h. The quartz tube containing the sample was sealed and transferred to a glovebox. Within the glovebox, about 100 mg of TiO_2_ sample was packed into the zirconia rotor, and one or two monolayers of IPA and water were introduced using micro syringes. The monolayer capacity was defined as the number of surface-exposed Ti sites quantified by NH_3_-TPD, assuming unimolecular binding. The required amount of IPA and H2O liquids were injected onto the samples inside NMR rotor in the glovebox via µL syringes. The rotor was sealed and then transferred to the NMR probe. NMR measurements were carried out at a sample spinning rate of 4 kHz to generate high resolution ^1^H and ^13^C NMR spectra. Under these conditions, the adsorbates were evenly distributed across the catalyst surface within a few minutes. A direct pulse sequence was used for acquiring ^1^H signals, consisting of a pulse width of 2.5 μs, an acquisition time of 100 ms and a recycle delay of 4 s. To generate ^13^C NMR signals, pulse width of 2.5 μs, an acquisition time of 100 ms and a recycle delay of 10 s were applied. ^1^H-^13^C cross polarization NMR was employed to investigate the TiO_2_ surface adsorbed species. A π/2 pulse width of 3.25 µs for ^1^H with a 2 s recycle delay and a contact time of 1 ms were applied for the latter measurements. All spectra were externally referenced to TMS (0 ppm) using adamantane as a second reference at 1.82 ppm for ^1^H and 38.48 ppm for ^13^C.^[66]^

**Catalysts evaluation****s**

Steady-state kinetic measurements and temperature programmed surface reactions (TPSR) were conducted on a fixed-bed quartz reactor of 9 mm i.d. that contains a coarse quartz frit at half-length for sample supporting. About 35 mg of catalysts, mixed with 300 mg of SiC (200 mesh, Sigma Aldrich) were loaded on the quartz frit, and the reactor was placed in a tube furnace. Bed temperature was controlled using a custom-built temperature controller coupled with a K-type thermocouple with its tip inserted to the center of the catalyst bed. Prior to activity measurements, the catalysts were pretreated at 450 °C for 1h in a 10% O_2_/He flow to remove water and other impurities. For steady-state kinetic measurements, IPA (Sigma Aldrich, >97 %) and deionized H_2_O at varying partial pressures were introduced to the catalyst using a He carrier gas (100 mL/min). To generate steady flows and partial pressures, IPA and H_2_O were introduced into vaporization zones located at the upstream of the reactor through gas tight syringes (Hamilton, Model 1002, 2.5 mL) mounted on syringe infusion pumps (KD Scientific, Model 100). The vaporization zones were heated to 90 °C for IPA and 120 °C for H_2_O. IPA and H_2_O partial pressures were adjusted by controlling the liquid injection rates of the syringe pumps. IPA and products were quantified using a gas chromatograph (Agilent, 7890A) equipped with a capillary column (Agilent HP-1, 19091Z-433, 30 m) connected to a flame ionization detector (FID). Prior to TPSR, the catalysts were first pretreated in a 10% O_2_/He flow and then cooled down to 120 °C. Then, 4 kPa IPA, 4 kPa H_2_O, or 10 kPa H_2_O:IPA mixture (3:2 partial pressure ratio) was introduced in (25 mL/min) in He at 120 °C and 1 atm for 20 min, followed by He purging or mixed with constant pressure of H_2_O. TPSR was conducted by heating the catalysts to 550 °C (10 °C min^-1^) in flowing He (25 mL/min) with H_2_O partial pressure varying from 0 to 8 kPa to study the impact of H_2_O. The desorption products were recorded with an Agilent Technologies brand quadrupole mass spectrometer. Turnover frequency of IPA dehydration is defined by the measured dehydration rate (r_propene_​) divided by the total moles of surface Ti sites per gram of catalyst (n_surface Ti sites_) obtained from NH_3_-TPD measurements with the assumption of a 1:1 stoichiometry between adsorbed NH_3_ and Ti surface sites

$TOF (s^{-1})=\frac{{r_{propene}(mol}_{propene} s^{-1} {g_{catalyst}}^{-1})}{n_{surface Ti sites}(mol {g_{catalyst}}^{-1})}$ (S1)

**Theoretical calculations**

The CP2K code was used to perform first-principles periodic DFT calculations.^[69]^ The valence electron wave functions were expanded in a double-basis set with polarization functions and an auxiliary plane wave basis set with an energy cutoff of 350 Ry.^[70]^ Perdew, Burke, and Enzerhof's (PBE) generalized gradient approximation exchange-correlation functional was used.^[71]^ Excess electrons associated with Ti 3d orbitals were treated with the DFT+U method with a U value of 7 eV, as previously suggested.^[72]^ When the maximum force convergence criteria of 4.5*10-5 Hartree/bohr were used in the geometry optimization, the maximum coordinate change of 3.0*104 bohr was achieved. Anatase TiO_2_(101) surfaces were used to model the TiO_2_ substrate, constructed with cell dimensions of 20.926×15.211×24.432 Å with 15 Å vacuum space to minimize the interaction between slabs. Anatase TiO_2_ (001) surface are represented here with cell dimensions of 19.361×19.365×23.633 Å. The Broyden-Fletcher-Goldfarb-Shanno (BGFS) algorithm was used to optimize each reaction state configuration,^[73]^ with self-consistent field (SCF) convergence criteria of 1.0*108 au. The DFT-D3 scheme with an empirical damped potential term was used to describe the van der Waals (vdW) dispersion interactions between adsorbates. The climbing image nudged elastic bands (CI-NEB) method with seven intermediate images along the reaction pathway between initial and final states was used to locate transition states of elementary steps^[74]^. Computational modeling of the NMR chemical shifts (DFT-NMR) was carried out using the Amsterdam Density Functional (ADF-2022) package to validate the detailed molecular interaction by directly comparing with experimental NMR results. Geometries were optimized using the Hybrid Becke−Lee−Yang−Parr-D3 functional on a cluster model extracted from the above periodic DFT calculations via CP2K. Basis sets were carried out by using the all-electron TZ2P basis set (triple-ζ, 2-polarization function) with Slater-type orbitals. NMR chemical shielding calculations were performed based on geometry-optimized structures at the same level of theory and with the same basis set for each atom. A reference with 10 molecules of IPA was established based on experimental chemical shifts of pure IPA. Scalar relativistic effects were accounted for using the zero-order regular approximation (ZORA). The models provided two layers of depth, where the bottom layer was frozen to preserve the crystal structure.^[75]^

1. **Notes**

**Note 1. Mass transfer calculation:**

We have evaluated the possibility of both internal and external mass transfer limitations under our reaction conditions.

1.1 Internal diffusion (Weisz–Prater):

$N_{WP}=\frac{r_{vol}R_{P}^{2}}{C_{A}D_{eff}}$ (S2A)

r_vol_ = observed rate per catalyst particle volume (mol m_cat_^-3^ s^-1^)

R_p_ = particle radius (m)

C_A_ = reactant concentration at particle surface

D_eff_ = effective diffusivity inside pores (m^2^ s^-1^)

On TiO_2_ (site density of 14 μmol g^-1^), when TOF of IPA dehydration is 0.15 s^-1^ at 533.15 K, 1 atm total pressure with 2 kPa IPA,

r_vol_ = 3.15 mol m^-3^ s^-1^

C_A_ = 0.451 mol m^-3^

D_eff_ = 10^-6^ m^2^ s^-1^

Crushed powder (~50 µm): N_WP_ = 0.0044

200 mesh (~75 µm sieve cut): N_WP_ = 0.0098

Both values are far below the threshold of 0.3, confirming no internal diffusion limitation.

1.2 External diffusion:

$Mears=\frac{r_{vol}R_{P}}{C_{A}k_{g}}$ (S2B)

$k_{g}$ = external gas-film mass-transfer coefficient (m s^-1^)

With typical gas properties at 533 K and ρ_g_ = 0.64 kg m^-3^, μ = 3.5 × 10^-5^ Pa s, D_AB_ = 1.0 × 10^-5^ m^2^ s^-1^, u = 0.10 m s^-1^

Powder (~50 µm): Mears = 3.5 × 10^-4^

200 mesh (~75 µm): Mears = 7.6×10^-4^

Both are ≪0.15, confirming no external film resistance.

For both crushed powder and 200-mesh catalyst fractions, the Weisz–Prater and Mears criteria are well below their thresholds, demonstrating that the reported rates reflect intrinsic kinetics rather than transport artifacts.

**Note 2. Facet analysis**

The quantitative facet analysis follows the approach reported in Nat. Commun. 8, 581 (2017), where the lengths of the lateral dimension (A) and the apical height (B) of the bipyramidal/nanosheet structures are measured from TEM images and used to calculate the areal fraction of (001) and (101) surfaces, using the equation of $Percentage\left( 001 \right)=\frac{cos68.3^{\circ}}{({\frac{A}{B})}^{2}+cos68.3^{\circ}-1}$. Representative TEM images of (A) nanosheet-shaped (001) particles and (B) bipyramidal (101) particles, together with facet ratio analysis, are shown in Figure S1. The detailed measurements are summarized in Table S1. For (101) particles, the average surface distribution is 7% (001) and 93% (101), while for (001) nanosheets, the average is 60% (001) and 40% (101). The low standard deviations (±0.02–0.07) confirm reproducibility across multiple measurements. These data were obtained from several independently synthesized batches during the course of this study, further supporting the robustness of the synthesis and analysis methods.

# Note 3. Extrapolation of Facet-Dependent Inhibition

3.1 Methodology
The inhibition ratio at 260 ^o^C is defined as:

$R=\frac{r_{dry}}{r_{PH_{2}O}}$ (S3)where the rates are selected at saturation IPA pressure, r_dry_ is the rate in the absence of water and $r_{PH_{2}O}$ is the rate at a given water partial pressure.

For a mixed-facet sample containing a fraction of (001) surface (denoted as *x*), the inhibition ratio is assumed to be a linear combination of pure-facet contributions R_(101)_ and R_(001)_:
 $R\left( x \right)=xR_{(001)}+\left( 1-x \right)R_{\left( 101 \right)}$ (S4)
From two measured samples with different (001) fractions, x = 0.6 and x = 0.07, we solve simultaneously:

$R\left( 0.6 \right)=0.6R_{(001)}+(1-0.6)R_{(101)}$ (S5)
 $R\left( 0.07 \right)=0.07R_{(001)}+(1-0.07)R_{(101)}$ (S6)
Combining eqs. S5 and S6 yields:
$R_{(001)}=\frac{0.93R\left( 0.6 \right)-0.4R(0.07)}{0.6-0.07}$ and $R_{(101)}=\frac{0.6R\left( 0.07 \right)-0.07R(0.6)}{0.6-0.07}$ at each given water partial pressure.

3.2 Worked example (2 kPa H_2_O)
For the (001)-enriched sample (R(0.6) = 3.5) and (101)-enriched sample (R(0.07) = 1.7):
 $R_{\left( 001 \right)}=\frac{0.93\times3.5-0.4\times1.7}{0.6-0.07}=4.86\approx4.9$ (S7)

$R_{(101)}=\frac{0.6\times1.7-0.07\times3.5}{0.6-0.07}=1.46\approx1.5$ (S8)

3.3 Results
Figure S2 shows the extrapolation of inhibition ratios as a function of (001) fraction, with separate curves for 2, 4, and 8 kPa H_2_O. The linear trends confirm that inhibition increases monotonically with (001) surface fraction, with maximum inhibition observed on pure (001) and minimum on pure (101).

**Note 4. Calculations of Enthalpy-Entropy Compensation**

The apparent discrepancy between the large increases in activation energies and the modest decreases in reaction rates can be rationalized by entropy–enthalpy compensation, as quantified below.

Transition-state theory (Eyring equation) is given as

$k_{i,j}=\frac{k_{B}T}{h}\exp\left( \frac{\Delta S_{i,j}^{\ddagger}}{R} \right)\exp\left( -\frac{\Delta H_{i,j}^{\ddagger}}{RT} \right)$ (S9)

where *k*_i,j_ denotes the rate constant of i (*i* = *m* or *n*, representing the monomeric or complex pathway, respectively) on the (j) facets (*j* = *001* or *101*). $\Delta H_{i,j}^{\ddagger}$ and $\Delta S_{i,j}^{\ddagger}$ are the activation enthalpy and entropy, respectively. *k*_B_ and *h* are the Boltzmann and Planck constants, respectively.

The rate constant ratio of complex to monomeric pathway on the (j) facets is

$\frac{k_{d,j}}{k_{m,j}}=\exp\left( \frac{\Delta S_{d,j}^{\ddagger}-\Delta S_{m,j}^{\ddagger}}{R} \right)\exp\left( -\frac{\Delta H_{d,j}^{\ddagger}-\Delta H_{m,j}^{\ddagger}}{RT} \right)=\exp\left( \frac{\Delta\left( \Delta S_{j}^{\ddagger} \right)}{R} \right)\exp\left( -\frac{\Delta\left( \Delta H_{j}^{\ddagger} \right)}{RT} \right)$ (S10)

where $\Delta\left( \Delta S_{j}^{\ddagger} \right)$ and $\Delta\left( \Delta H_{j}^{\ddagger} \right)$ are the differences between activation entropies ($\Delta S_{d,j}^{\ddagger}-\Delta S_{m,j}^{\ddagger}$), and between activation enthalpies ($\Delta H_{d,j}^{\ddagger}-\Delta H_{m,j}^{\ddagger}$) of complex and monomeric pathways. Since the relationship between activation barrier and enthalpy of activation is

$E_{a,i,j}=\Delta H_{i,j}^{\ddagger}+RT$ (S11)

Substituting eq. S11 into S10 gives

$\frac{k_{d,j}}{k_{m,j}}=\exp\left( \frac{\Delta\left( \Delta S_{j}^{\ddagger} \right)}{R} \right)\exp\left( -\frac{E_{a,d,j}-E_{a,m,j}}{RT} \right)=\exp\left( \frac{\Delta\left( \Delta S_{j}^{\ddagger} \right)}{R} \right)\exp\left( -\frac{\Delta E_{a,j}}{RT} \right)$ (S12)

where $\Delta E_{a,j}$ is the differences between activation barriers of complex and monomeric pathways ($E_{a,d,j}-E_{a,m,j}$). Taking the natural logarithm of eq. S12 gives

$ln\left( \frac{k_{d,j}}{k_{m,j}} \right)=\frac{\Delta\left( \Delta S_{j}^{\ddagger} \right)}{R}-\frac{\Delta E_{a,j}}{RT}$ (S13)

On TiO_2_(001), the activation barrier of IPA dehydration at 1 atm total pressure with 1 kPa IPA and 0 kPa H_2_O is 142 kJ mol^-1^ (Figure 2E). Since the reaction in absence of water could only proceed via the monomeric pathway, $E_{a,m,001}$ equals to 142 kJ mol^-1^. On the other hand, the activation barrier at 1 atm total pressure with 1 kPa IPA and 12 kPa H_2_O is 182 ± 4 kJ mol^-1^. According to the parameters in Table S3, the rate ratio of complex to monomeric pathway is 5.7 at 260 ^o^C, 1 atm total pressure with 1 kPa IPA and 12 kPa H_2_O, indicating negligible contribution from monomeric pathway; and thus $E_{a,d,001}$ approximately equals 182 ± 5 kJ mol^-1^. Therefore,

$\Delta E_{a,001}=\Delta\left( \Delta H_{i,j}^{\ddagger} \right)=40 kJ {mol}^{-1}$ (S14)

Besides, k_d,001_ and k_m,001_ are 2.49 (±0.15) × 10^-3^ and 1.56 (±0.17) × 10^-2^ s^-1^, respectively (260 ^o^C, Table S5). Substituting k_d,001_, k_m,001_ and $\Delta E_{a,001}$ into eq. 13 gives

$\Delta\left( \Delta S_{001}^{\ddagger} \right)=R\left[ \ln\left( \frac{k_{d,j}}{k_{m,j}} \right)+\frac{\Delta E_{a,j}}{RT} \right]=60 J {mol}^{-1}K⁻¹$ (S15)

Likewise, $E_{a,m,101}$ and $E_{a,d,101}$ on TiO_2_(101) are approximately 135 ± 1 and 160 ± 2 kJ mol^-1^, respectively, as shown in Figure 2F, which give $\Delta E_{a,101}$ of 25 kJ mol^-1^. Since k_d,101_ and k_m,101_ are 7.10 (±0.90) × 10^-3^ s^-1^ and 2.87 (±0.03) × 10^-2^ s^-1^ (260 ^o^C, Table S3), respectively, $\Delta\left( \Delta S_{101}^{\ddagger} \right)$ equals to 35 J mol^-1^ K^-1^.

# Note 5. Derivation of Turnover Rate Expression

Tables S1-S2 demonstrate the elementary steps of IPA dehydration on TiO_2_(001) and (101), respectively. As described in the main manuscript, E2 eliminations of Ti(*i*-OC_3_H_7_)-O_s_(H)-Ti(OH) (**Step (001)-m5**) and Ti(*i*-OC_3_H_7_···H_2_O)-O_s_(H)-Ti(OH) (**Step (001)-d5**) are the kinetically relevant steps for monomeric and complex pathways on TiO_2_(001), respectively. Thus, the pseudo steady-state approximation of surface species leads to the following rate expression, in which r_001_ is the sum of turnover rates of monomeric (r_m,001_) and complex pathways (r_d,001_),

$r_{001}=r_{m,001}+r_{d,001}$

$=k_{m,001}\theta_{Ti\left( i-OC_{3}H_{7} \right)-O_{s}(H)-Ti(OH)}+k_{d,001}\theta_{Ti\left( i-{OC_{3}H_{7}\cdots H}_{2}O \right)-O_{s}(H)-Ti(OH)}$ (S16)

The fractional coverages of surface species, denoted as θ*_γ_*, (*γ* = Ti(*i*-OC_3_H_7_)-O_s_(H)-Ti(OH), Ti(H_2_O)-O_s_-Ti(OH), Ti(H_2_O)_2_-O_s_-Ti(OH), and Ti(*i*-OC_3_H_7_···H_2_O)-O_s_(H)-Ti(OH), etc.) are given as

$\theta_{Ti\left( i-OC_{3}H_{7} \right)-O_{s}(H)-Ti(OH)}=K_{I,001}[IPA]\theta_{Ti-O_{s}-Ti(OH)}$ (S17)

$\theta_{Ti\left( H_{2}O \right)-O_{s}-Ti(OH)}=K_{W1,001}[H_{2}O]\theta_{Ti-O_{s}-Ti(OH)}$ (S18)

$\theta_{Ti{{(H}_{2}O)}_{2}-O_{s}-Ti\left( OH \right)}=K_{W1-2,001}K_{W1,001}\left[ H_{2}O \right]^{2}\theta_{Ti-O_{s}-Ti\left( OH \right)}$

$=K_{W2,001}{[H_{2}O]}^{2}\theta_{Ti-O_{s}-Ti(OH)}$ (S19)

where $K_{W2,001}$ is the overall equilibrium constant for **Steps (001)-2** and **(001)-4**.

$\theta_{Ti\left( i-{OC_{3}H_{7}\cdots H}_{2}O \right)-O_{s}\left( H \right)-Ti\left( OH \right)}=K_{W\cdot D,001}\left[ H_{2}O \right]\theta_{Ti\left( i-OC_{3}H_{7} \right)-O_{s}\left( H \right)-Ti\left( OH \right)}$

$=K_{W\cdot D,001}K_{I,001}[IPA][H_{2}O]\theta_{Ti-O_{s}-Ti(OH)}$ (S20)

The total fractional coverage of surface species and exposed active sites is

$\theta_{Ti-O_{s}-Ti(OH)}+\theta_{Ti\left( i-OC_{3}H_{7} \right)-O_{s}(H)-Ti(OH)}+\theta_{Ti\left( H_{2}O \right)-O_{s}-Ti(OH)}+\theta_{Ti{{(H}_{2}O)}_{2}-O_{s}-Ti(OH)}+\theta_{Ti\left( {i-OC_{3}H_{7}\cdots H}_{2}O \right)-O_{s}(H)-Ti(OH)}=1$ (S21)

Substituting eqs. S17-S20 into eq. S21 gives

$\theta_{Ti-O_{s}-Ti(OH)}=\frac{1}{1+K_{I,001}\left[ IPA \right]+K_{W1,001}\left[ H_{2}O \right]+K_{W2,001}{[H_{2}O]}^{2}+K_{W\cdot D,001}K_{I,001}[IPA][H_{2}O]}$ (S22)

The combination of eqs. S17, S20, S21 and S16 yields

$r_{001}=\theta_{Ti-O_{s}-Ti(OH)}\left( k_{m,001}K_{I,001}[IPA]+k_{d,001}K_{W\cdot D,001}K_{I,001}[IPA][H_{2}O] \right)$

$=\frac{k_{m,001}K_{I,001}[IPA]+k_{d,001}K_{W\cdot D,001}K_{I,001}[IPA][H_{2}O]}{1+K_{I,001}\left[ IPA \right]+K_{W1,001}\left[ H_{2}O \right]+K_{W2,001}{[H_{2}O]}^{2}+K_{W\cdot D,001}K_{I,001}[IPA][H_{2}O]}$ (S23)

Likewise, since **Steps (101)-m-5** and **(101)-d-5** are the kinetically relevant steps for monomeric and complex pathways on TiO_2_(101), respectively, the total turnover rate on TiO_2_(101) is

$r_{101}=r_{m,101}+r_{d,101}=k_{m,101}\theta_{Ti\left( IPA \right)-O_{s}}+k_{d,101}\theta_{Ti\left( IPA{\cdots H}_{2}O \right)-O_{s}}$ (S24)

The fractional coverages of surface species are

$\theta_{Ti\left( IPA \right)-O_{s}}=K_{I,101}[IPA]\theta_{Ti-O_{s}}$ (S25)

$\theta_{Ti\left( H_{2}O \right)-O_{s}}=K_{W1,101}[H_{2}O]\theta_{Ti-O_{s}}$ (S26)

$\theta_{Ti{{(H}_{2}O)}_{2}-O_{s}}=K_{W1-2,101}K_{W1,101}{[H_{2}O]}^{2}\theta_{Ti-O_{s}}=K_{W2,101}{[H_{2}O]}^{2}\theta_{Ti-O_{s}}$ (S27)

where $K_{W2,101}$ is the overall equilibrium constant for **Steps (101)-2** and **(101)-4**.

$\theta_{Ti\left( IPA{\cdots H}_{2}O \right)-O_{s}}=K_{W\cdot D,101}\left[ H_{2}O \right]\theta_{Ti\left( IPA \right)-O_{s}}$

$=K_{W\cdot D,101}K_{I,101}[IPA][H_{2}O]\theta_{Ti-O_{s}}$ (S28)

The sum of total fractional coverages is given as

$\theta_{Ti-O_{s}}+\theta_{Ti\left( IPA \right)-O_{s}}+\theta_{Ti\left( H_{2}O \right)-O_{s}}+\theta_{Ti{{(H}_{2}O)}_{2}-O_{s}}+\theta_{Ti\left( IPA{\cdots H}_{2}O \right)-O_{s}}=1$ (S29)

Substituting eqs. S25-S28 into eq. S29 leads to

$\theta_{Ti-O_{s}}\left( 1+K_{I,101}\left[ IPA \right]+K_{W1,101}\left[ H_{2}O \right]+K_{W2,101}{[H_{2}O]}^{2}+K_{W\cdot D,101}K_{I,101}[IPA][H_{2}O] \right)=1$ (S30)

which can be rearranged into

$\theta_{Ti-O_{s}}=\frac{1}{1+K_{I,101}\left[ IPA \right]+K_{W1,101}\left[ H_{2}O \right]+K_{W2,101}{[H_{2}O]}^{2}+K_{W\cdot D,101}K_{I,101}[IPA][H_{2}O]}$ (S31)

Thus, substituting eqs. S25, S28 and S30 into eq. S24 generates

$r_{101}=\frac{k_{m,101}K_{I,101}[IPA]+k_{d,101}K_{W\cdot D,101}K_{I,101}[IPA][H_{2}O]}{1+K_{I,101}\left[ IPA \right]+K_{W1,101}\left[ H_{2}O \right]+K_{W2,101}{[H_{2}O]}^{2}+K_{W\cdot D,101}K_{I,101}[IPA][H_{2}O]}$ (S32)

Thus, the general expression for IPA dehydration on TiO_2_(001) (eq. S23) and TiO_2_(101) (eq. S32) can be given as

$r_{j}=\frac{k_{m,j}K_{I,j}[IPA]+k_{d,j}K_{W\cdot D,j}K_{I,j}[IPA][H_{2}O]}{1+K_{I,j}\left[ IPA \right]+K_{W1,j}\left[ H_{2}O \right]+K_{W2,j}{[H_{2}O]}^{2}+K_{W\cdot D,j}K_{I,j}[IPA][H_{2}O]}$ (S33)

which is eq. 1 in the main manuscript.

1. **Figures**


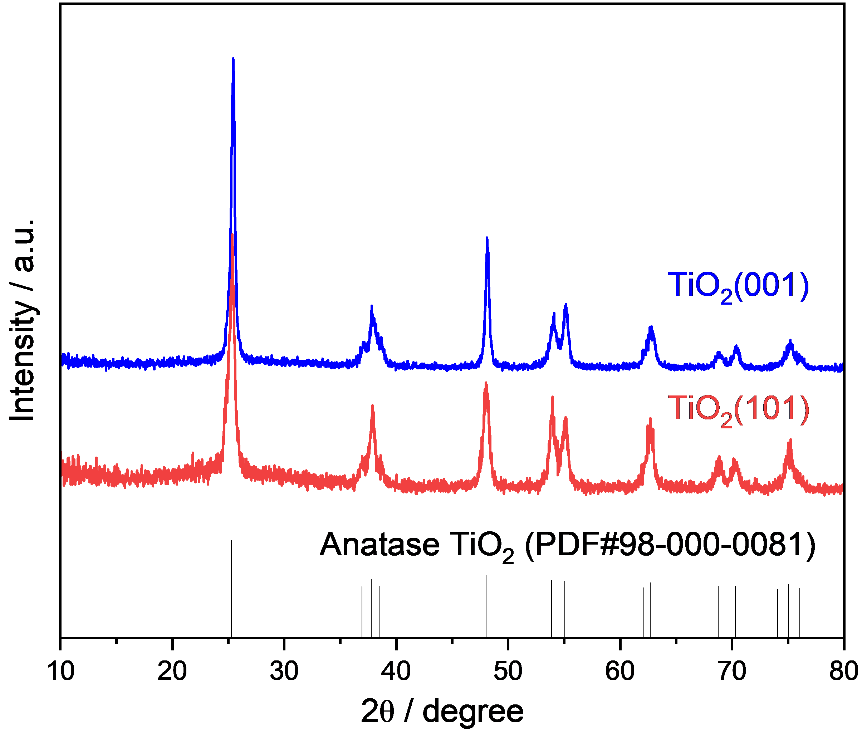


Figure S1. XRD patterns of fresh TiO_2_(001) and (101) samples.


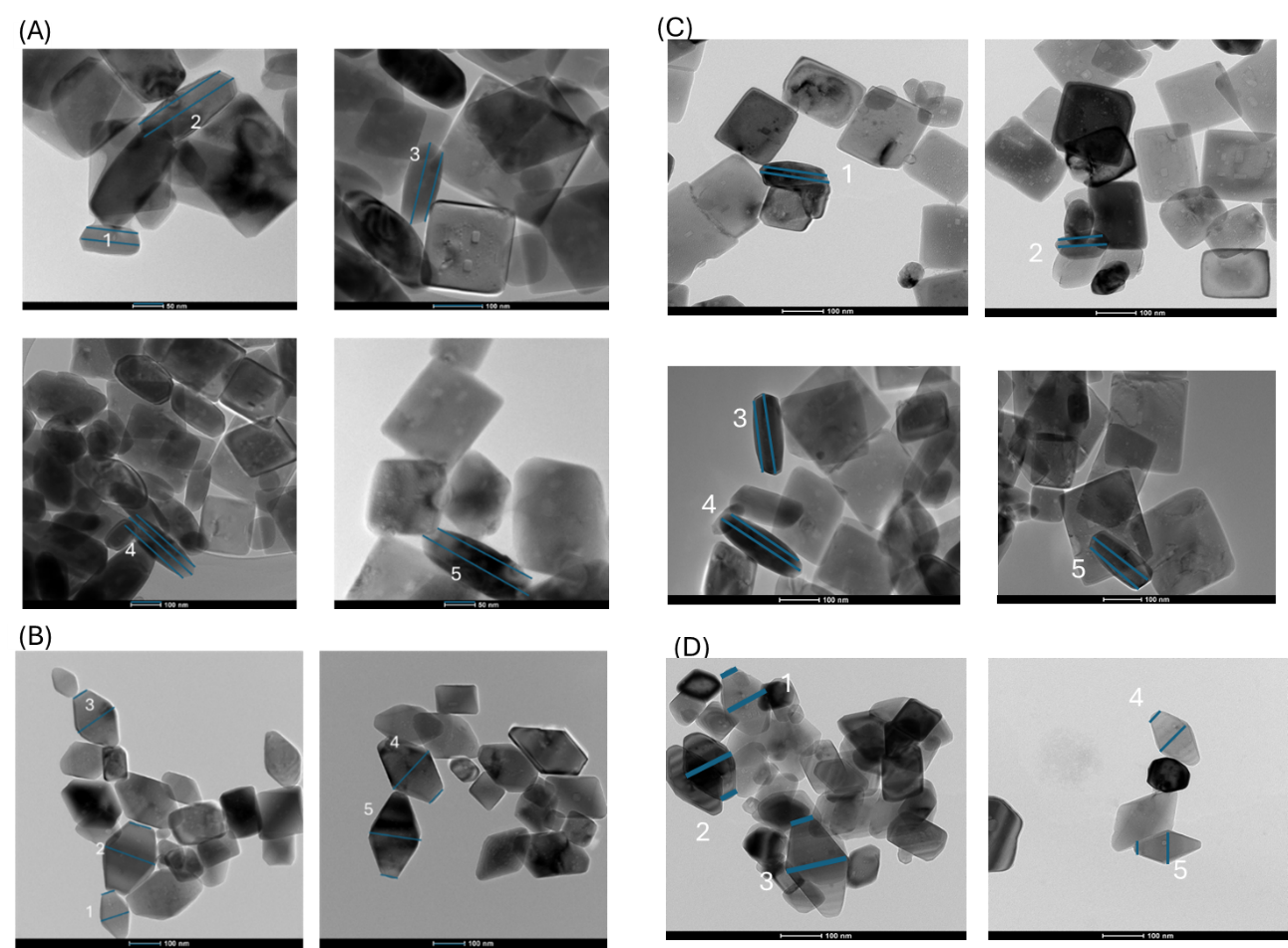


Figure S2. TEM images of (A) fresh TiO_2_(001) with nanosheet shapes and (B) fresh TiO_2_(101) with bipyramidal shapes. (C) and (D) TEM images of (001) and (101) after reaction with nanosheet shapes and bipyramidal shapes preserved respectively. Blue lines are dimensions of facets and are used to calculate the fraction of (001) and (101) surfaces.


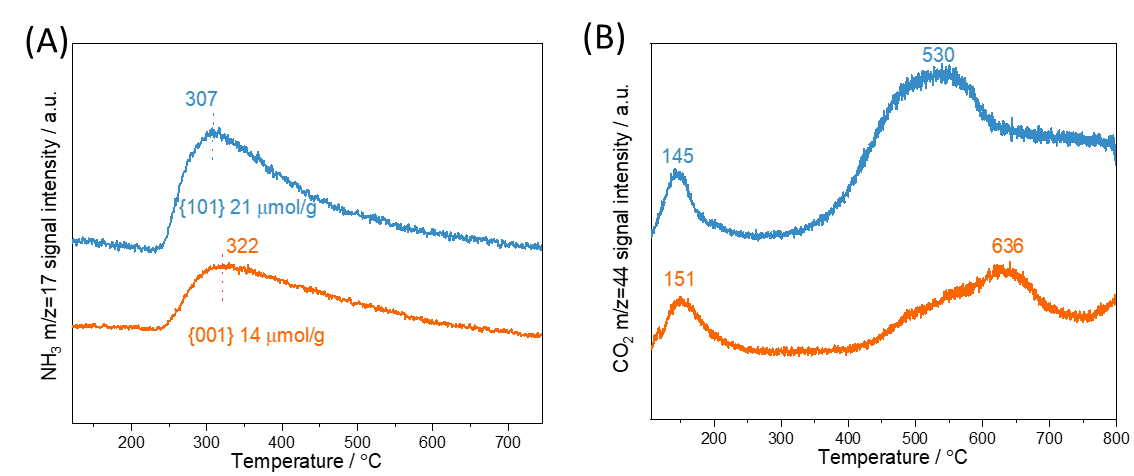


Figure S3 (A) NH_3_-TPD and (B) CO_2_-TPD profiles of TiO_2_(101) and (001). Operating conditions: samples were pretreated at 450 °C for 1 h under He (50 mL min^-1^), cooled to 100 °C, and then saturated with 1% NH_3_/He or 10% CO_2_/He (50 mL min^-1^). Temperature was then ramped to 700 °C at 10 °C min^-1^ under He (50 mL min^-1^).


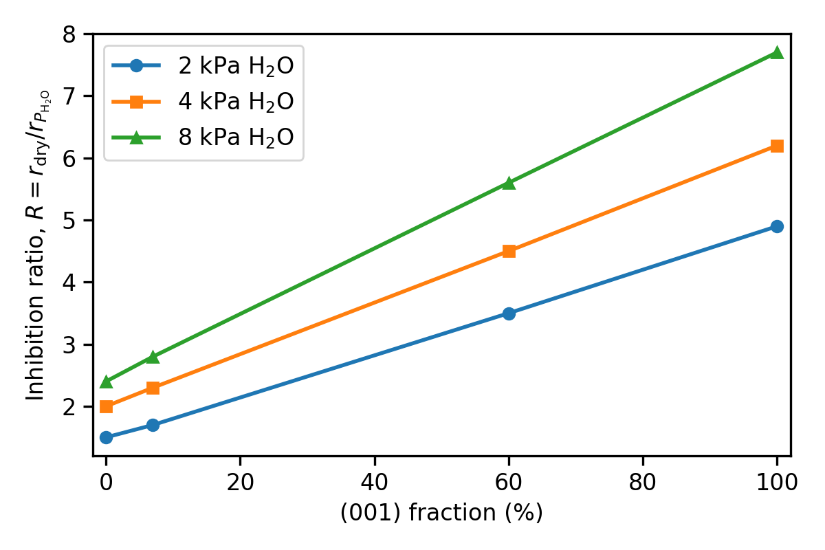


Figure S4. Extrapolation of inhibition ratios vs. (001) fraction at different water pressures (2, 4, 8 kPa).


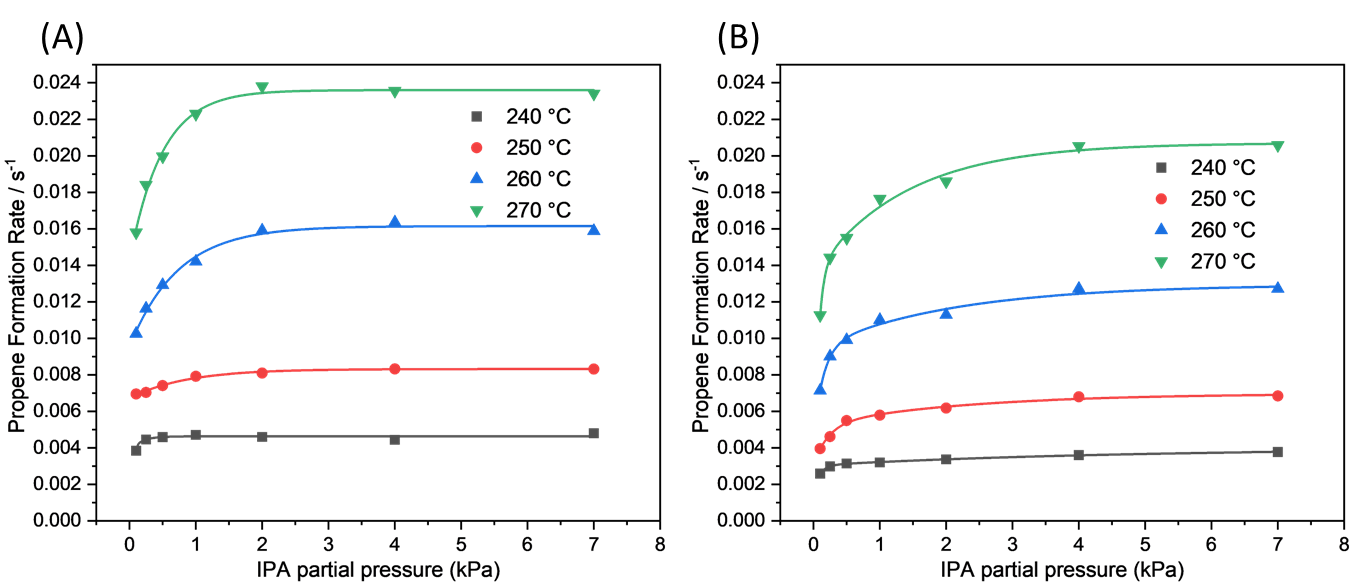


Figure S5. Kinetic dependence of IPA dehydration on TiO_2_(001) (A) in the absence of water and (B) in the presence of water (0.2 kPa water). Operating conditions: 240-270 ^o^C,1 atm total pressure with 0.1-7 kPa IPA, 0.2 kPa co-fed H_2_O, and balance He, and 47.6 cm^3^ g^-1^ s^-1^ GHSV.


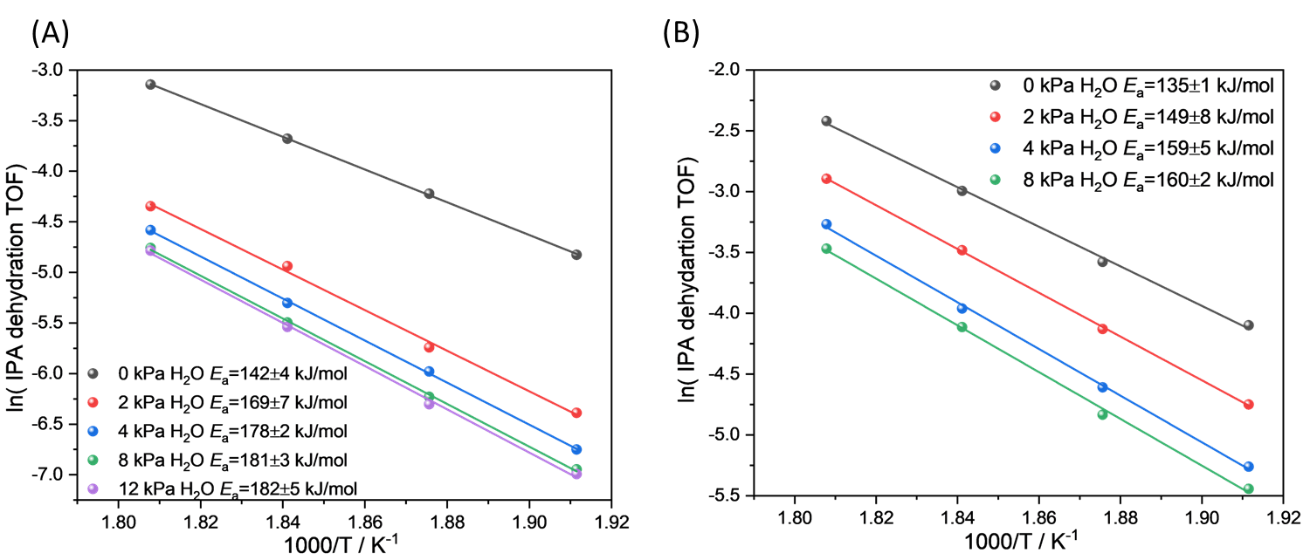


Figure S6. Arrhenius plots of IPA dehydration on (A) TiO_2_(001) and (B) (101) at 1 atm total pressure with 1 kPa IPA, 0-12 kPa co-fed H_2_O and 47.6 cm^3^ g^-1^ s^-1^ GHSV.

Figure S7. High vacuum IR by introducing IPA and H_2_O with increasing pressure from 0.1 mbar to 1.1 mbar on (101) at 100 °C.


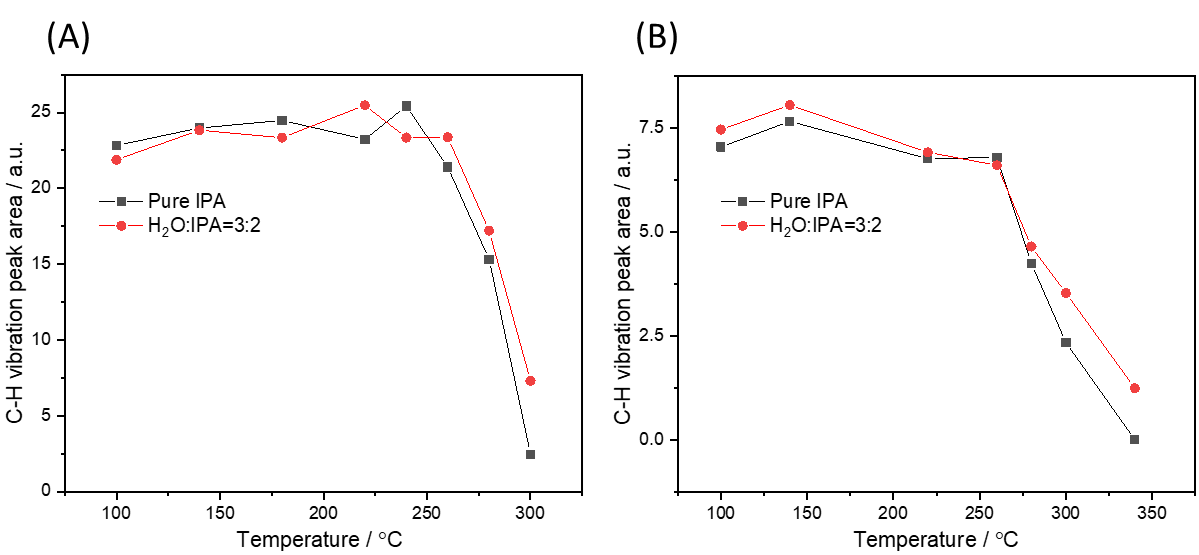


Figure S8. C-H vibration peak area integrated from DRIFTS spectra versus temperature with pure IPA adsorption and H_2_O: IPA partial pressure ratio of 3:2 on (A) (101) and (B) (001)

Figure S9. DRIFTS spectra of -OH region with (bottom) pure IPA on (101) and (top) H_2_O&IPA on (101).


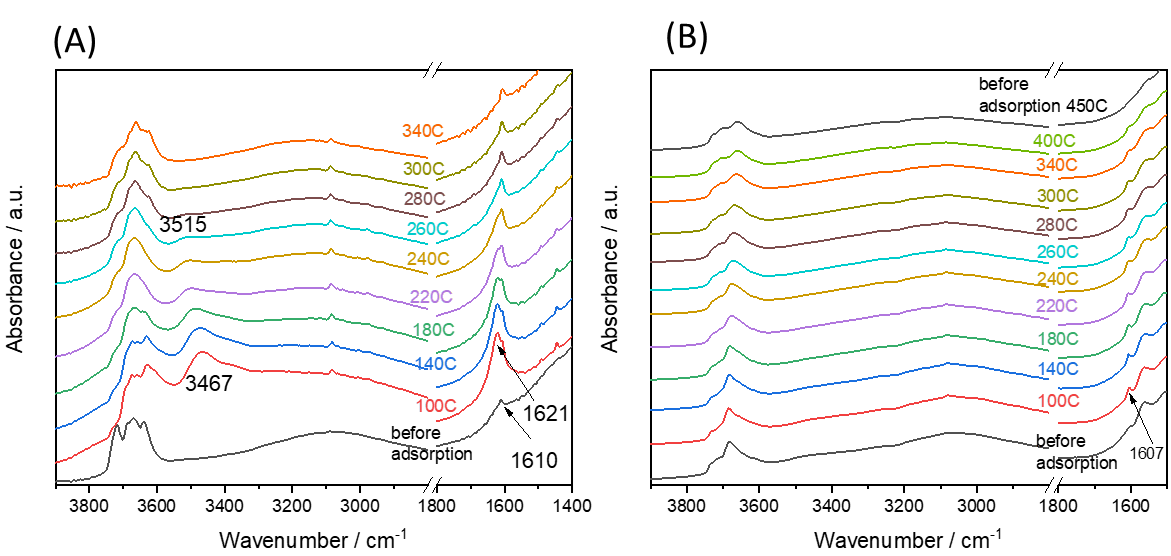


Figure S10. (A) and (B) in situ DRIFTS-TPD spectra of adsorption and desorption of pure H_2_O on (101) and (001). Operating conditions: catalysts (20 mg) were pretreated in 10% O_2_/He at 450 °C at ambient pressure for 1 h, and then treated with H_2_O in flowing He at 100 °C until saturation, followed by ramping from 100 °C to 450 °C (10 °C min^-1^) in flowing He (50 mL min^-1^).


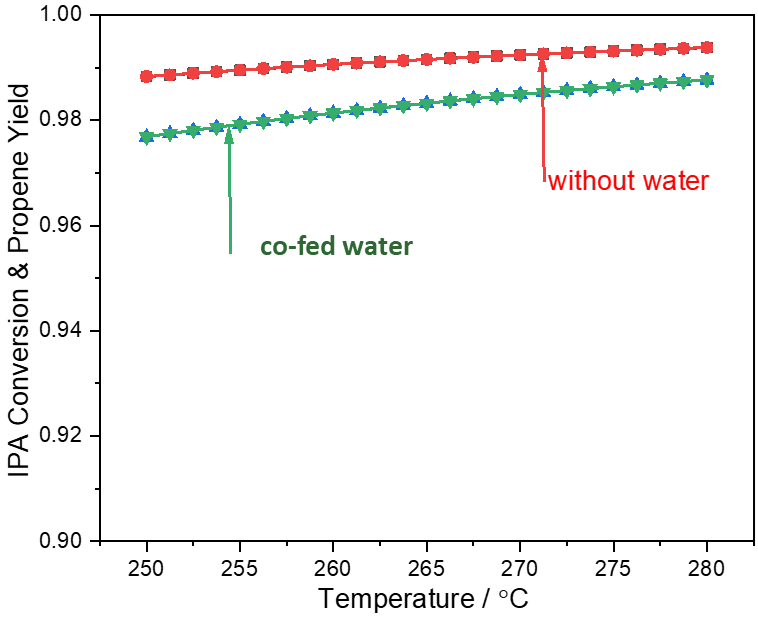


Figure S11. Thermodynamic equilibria calculations of IPA dehydration at temperature range from 250-280 °C with or without co-fed water (water:IPA = 4:1 or 0:1, respectively)


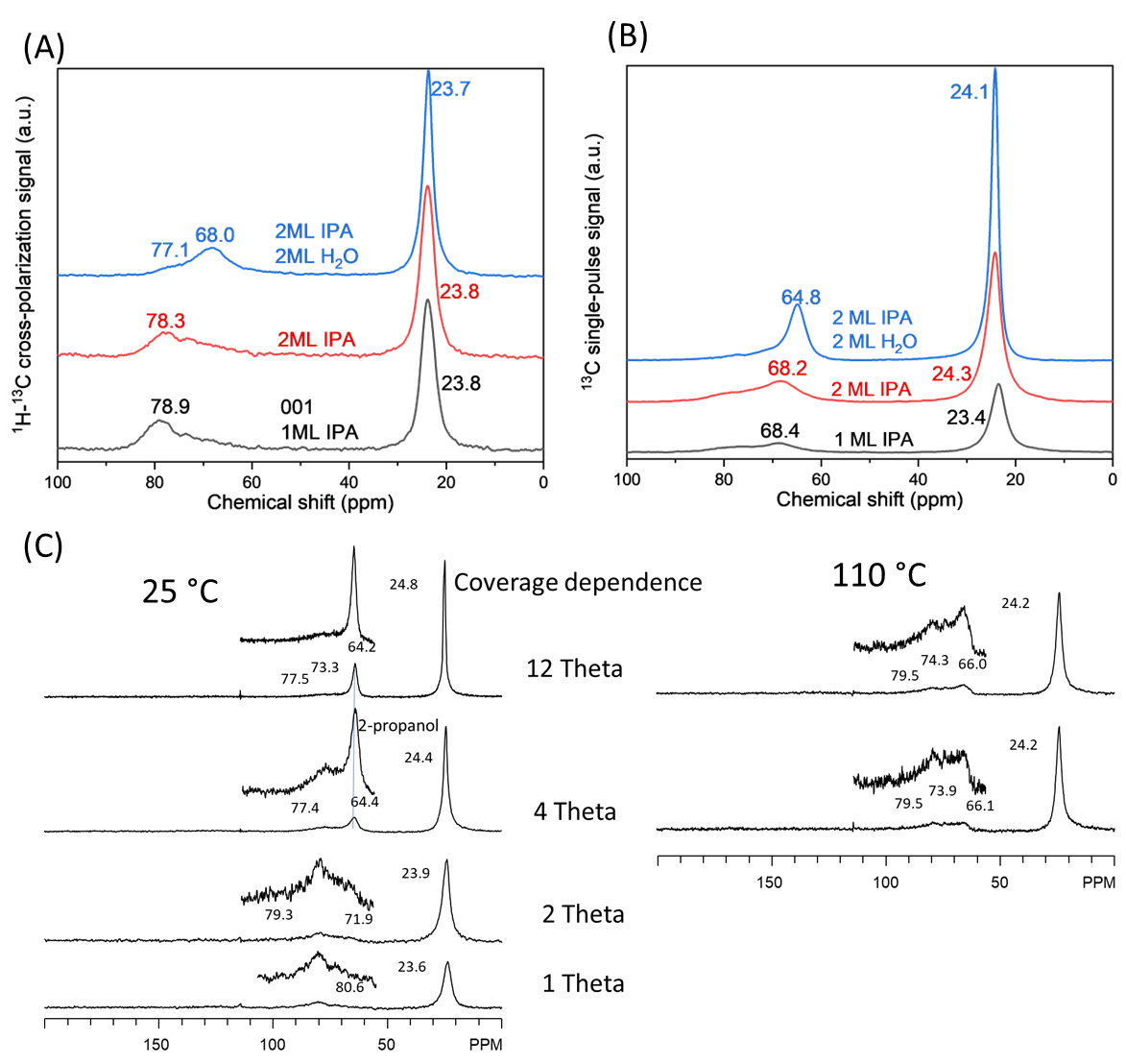


Figure S12. *In situ* ^13^C direct polarization NMR of IPA and water adsorbed on (A) (001) and (B) (101); (C)^13^C NMR of different coverages IPA on (001) at 25 °C(DP) and 110 °C(CP)


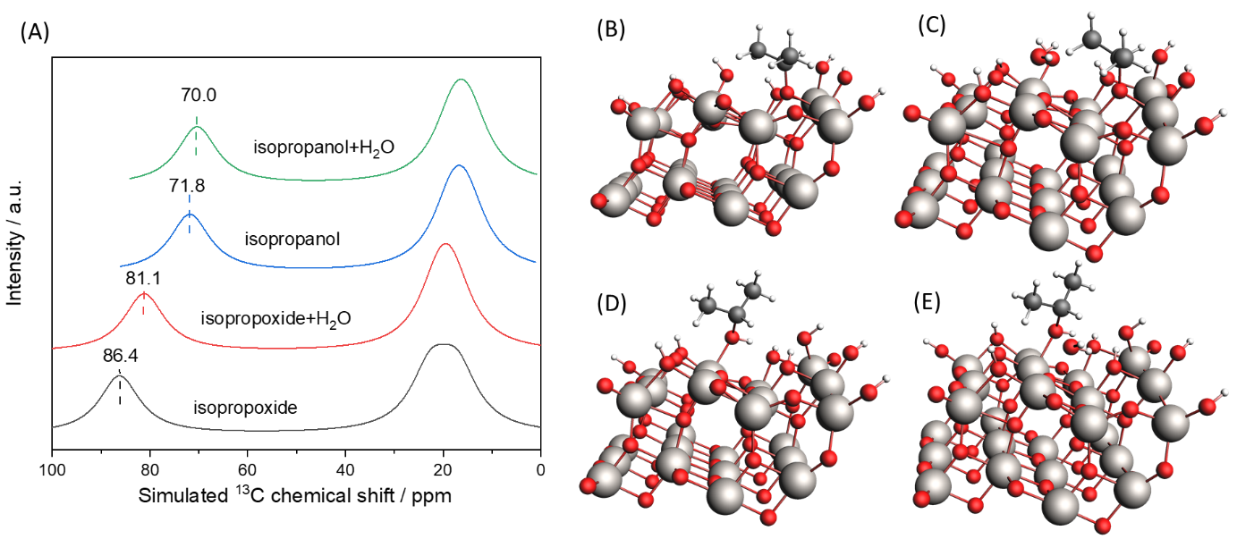


Figure S13. (A) DFT-NMR simulated ^13^C NMR spectra of (B) isopropoxide, (C) isopropoxide + H_2_O, (D)isopropanol, and (E)isopropanol + H_2_O adsorbed on TiO_2_.


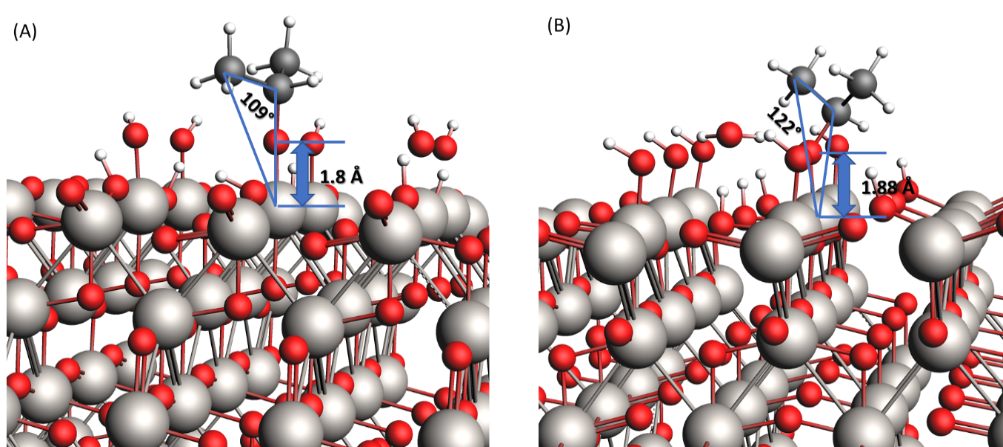


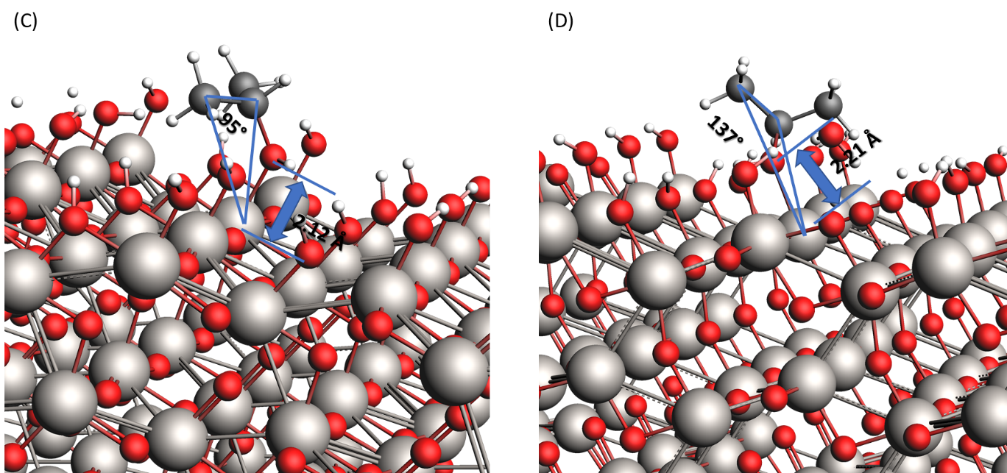


Figure S14. Zoom in model structures illustrating bond angles and bond distances with (A) IPA on TiO_2_ (001), (B) IPA+H_2_O on TiO_2_ (001), (C) IPA on TiO_2_ (101) and (D) IPA+H_2_O on TiO_2_ (101).


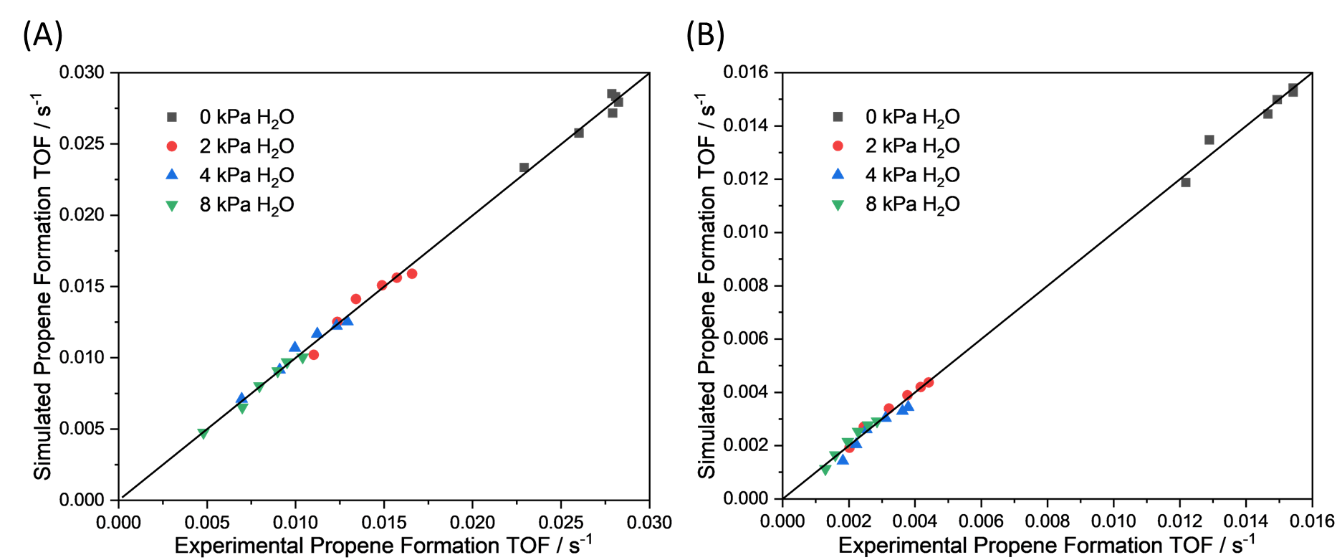


Figure S15. Parity plots for the predicted (from eq. S33 and parameters in Table S7) and measured turnover frequencies on (A) TiO_2_(101) and (B) (001) at 260 °C, 1 atm total pressure with 0.25-8 kPa IPA, 0-8 kPa H_2_O, balance He, and 47.6 cm^3^ g^-1^ s^-1^ GHSV. The solid line represents a perfect prediction of turnover frequencies for propene formation

Sensitivity analyses for the parameters in eq. 1 were performed by varying the optimal values by ±25% and then comparing the sum of squared residuals between the predicted and experimental data. As shown in Figures S16 and 17, the parameters are sensitive to the changes, confirming the reliability of the predicted values.


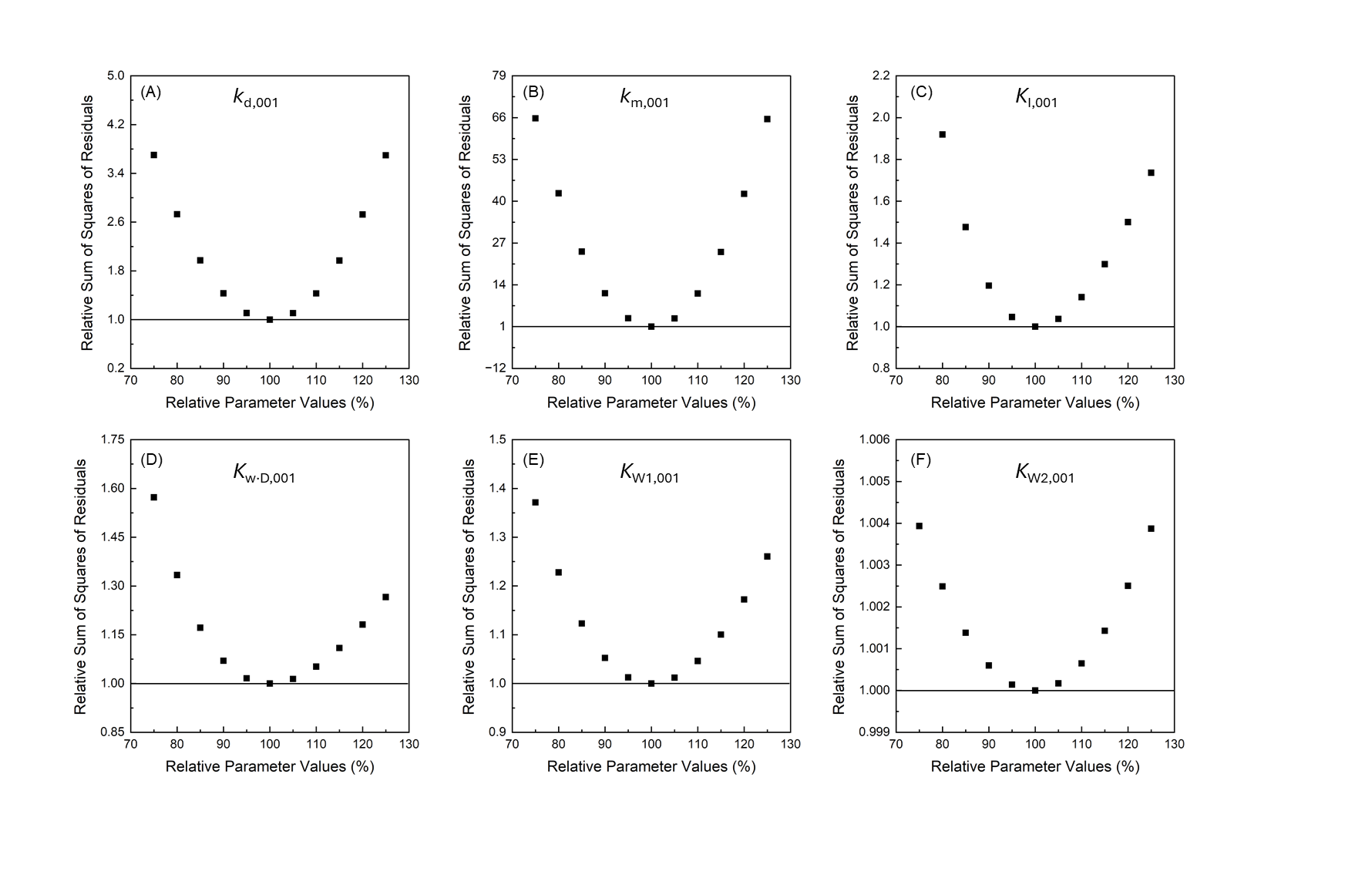


Figure S16. Sensitivity of sum of squares of residuals to variations in optimal kinetic and thermodynamic parameters of the rate expression (Table S3, eq. S33) on TiO_2_ (001) at 260 ^o^C,1 atm total pressure with 0.25-8 kPa IPA, 0-8 kPa H_2_O, and balance He, and 47.6 cm^3^ g^-1^ s^-1^ GHSV.


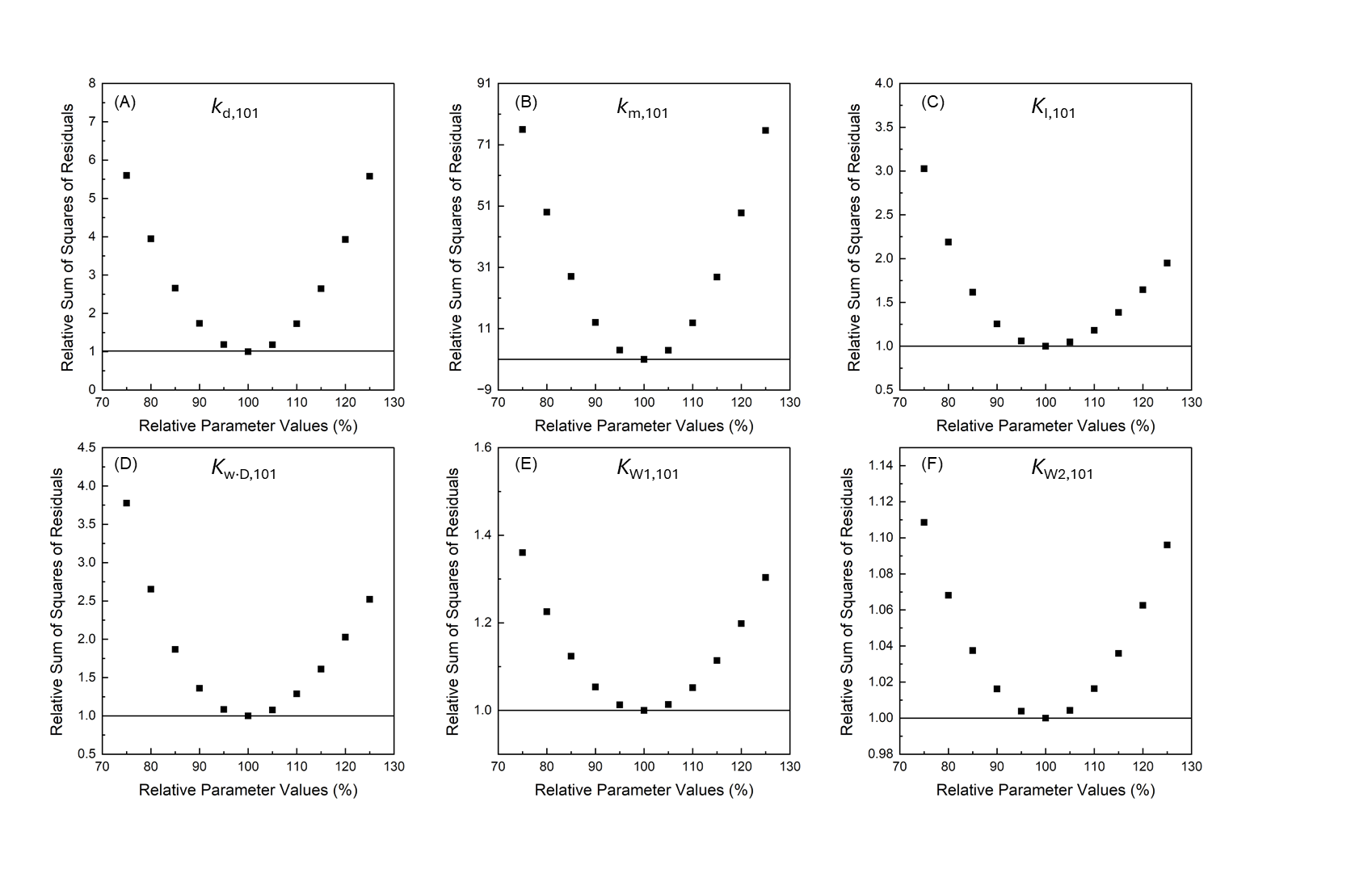


Figure S17. Sensitivity of sum of squares of residuals to variations in optimal kinetic and thermodynamic parameters of the rate expression (Table S3. eq. S33) on TiO_2_ (101) at 260 ^o^C,1 atm total pressure with 0.25-8 kPa IPA, 0-8 kPa H_2_O, and balance He, and 47.6 cm^3^ g^-1^ s^-1^ GHSV.

1. **Tables**

Table S1. Length measurement and (001) percentage calculations

|  | A length | B length | "%001" | "%101" |
| --- | --- | --- | --- | --- |
| (101) |  |  |  |  |
| 1 | 0.411096 | 0.199249 | 0.101952033 | 0.898048 |
| 2 | 0.746726 | 0.303645 | 0.068250829 | 0.9317492 |
| 3 | 0.62426 | 0.238537 | 0.059458157 | 0.9405418 |
| 4 | 0.738241 | 0.245153 | 0.043819416 | 0.9561806 |
| 5 | 0.742765 | 0.275862 | 0.055857893 | 0.9441421 |
|  |  | Average | 0.07 | 0.93 |
|  |  | Standard deviation | 0.02 | 0.02 |
| (001) |  |  |  |  |
| 1 | 0.977394 | 0.867006 | 0.577188653 | 0.4228113 |
| 2 | 1.828579 | 1.670479 | 0.650973145 | 0.3490269 |
| 3 | 1.402462 | 1.203038 | 0.507364167 | 0.4926358 |
| 4 | 1.343801 | 1.244829 | 0.691012433 | 0.3089876 |
| 5 | 1.905387 | 1.669641 | 0.550157117 | 0.4498429 |
|  |  | Average | 0.60 | 0.40 |
|  |  | Standard deviation | 0.07 | 0.07 |
|  |  |  |  |  |

Table S2. Turnover frequencies and conversions of IPA dehydration on TiO_2_(001) at 260 ^o^C, 1 atm total pressure with 0.25-8 kPa IPA, 0-8 kPa H_2_O, balance He, and 47.6 cm^3^ g^-1^ s^-1^ GHSV.

| Partial Pressure, kPa | | TOF, s^-1^ | Conversion, % | Generated Waer, kPa |
| --- | --- | --- | --- | --- |
| IPA, kPa | H_2_O, kPa |  |  |  |
| 0.25 | 0 | 0.01218 | 3.55 | 0.009 |
| 0.5 | 0 | 0.01289 | 1.88 | 0.009 |
| 1 | 0 | 0.01466 | 1.07 | 0.011 |
| 2 | 0 | 0.01494 | 0.54 | 0.011 |
| 4 | 0 | 0.01542 | 0.28 | 0.011 |
| 8 | 0 | 0.01542 | 0.14 | 0.011 |
| 0.25 | 2 | 0.00202 | 0.59 | 0.001 |
| 0.5 | 2 | 0.00245 | 0.36 | 0.002 |
| 1 | 2 | 0.00321 | 0.23 | 0.002 |
| 2 | 2 | 0.00377 | 0.14 | 0.003 |
| 4 | 2 | 0.00417 | 0.08 | 0.003 |
| 8 | 2 | 0.00441 | 0.04 | 0.003 |
| 0.25 | 4 | 0.00182 | 0.53 | 0.001 |
| 0.5 | 4 | 0.00222 | 0.32 | 0.002 |
| 1 | 4 | 0.00253 | 0.18 | 0.002 |
| 2 | 4 | 0.00311 | 0.11 | 0.002 |
| 4 | 4 | 0.00362 | 0.07 | 0.003 |
| 8 | 4 | 0.00379 | 0.03 | 0.003 |
| 0.25 | 8 | 0.00129 | 0.38 | 0.001 |
| 0.5 | 8 | 0.00159 | 0.23 | 0.001 |
| 1 | 8 | 0.00197 | 0.14 | 0.001 |
| 2 | 8 | 0.00228 | 0.08 | 0.002 |
| 4 | 8 | 0.00258 | 0.05 | 0.002 |
| 8 | 8 | 0.00285 | 0.03 | 0.002 |

Table S3. Turnover frequencies and conversions of IPA dehydration on TiO_2_(101) at 260 ^o^C, 1 atm total pressure with 0.25-8 kPa IPA, 0-8 kPa H_2_O, balance He, and 47.6 cm^3^ g^-1^ s^-1^ GHSV.

| Partial Pressure, kPa | | TOF, s^-1^ | Conversion, % | Generated Waer, kPa |
| --- | --- | --- | --- | --- |
| IPA | H_2_O |  |  |  |
| 0.25 | 0 | 0.02292 | 10.02 | 0.025 |
| 0.5 | 0 | 0.02602 | 5.69 | 0.028 |
| 1 | 0 | 0.02792 | 3.05 | 0.031 |
| 2 | 0 | 0.02825 | 1.54 | 0.031 |
| 4 | 0 | 0.02809 | 0.77 | 0.031 |
| 8 | 0 | 0.02787 | 0.38 | 0.03 |
| 0.25 | 2 | 0.01103 | 4.82 | 0.012 |
| 0.5 | 2 | 0.01236 | 2.7 | 0.014 |
| 1 | 2 | 0.01341 | 1.47 | 0.015 |
| 2 | 2 | 0.01489 | 0.81 | 0.016 |
| 4 | 2 | 0.01572 | 0.43 | 0.017 |
| 8 | 2 | 0.01658 | 0.23 | 0.018 |
| 0.25 | 4 | 0.00695 | 3.04 | 0.008 |
| 0.5 | 4 | 0.0091 | 1.99 | 0.01 |
| 1 | 4 | 0.00996 | 1.09 | 0.011 |
| 2 | 4 | 0.01122 | 0.61 | 0.012 |
| 4 | 4 | 0.01231 | 0.34 | 0.013 |
| 8 | 4 | 0.01294 | 0.18 | 0.014 |
| 0.25 | 8 | 0.00479 | 2.09 | 0.005 |
| 0.5 | 8 | 0.00698 | 1.53 | 0.008 |
| 1 | 8 | 0.00796 | 0.87 | 0.009 |
| 2 | 8 | 0.00899 | 0.49 | 0.01 |
| 4 | 8 | 0.00952 | 0.26 | 0.01 |
| 8 | 8 | 0.0104 | 0.14 | 0.011 |

Table S4. Kinetic isotope effect of IPA dehydration using CD_3_CH(OH)CD_3_ or CH_3_CH(OD)CH_3_ with co-fed water on TiO_2_(001) and (101) at 260 ^o^C. 1 atm total pressure with 1 kPa reactant, 4 kPa H_2_O, and balance He, and 47.6 cm^3^ g^-1^ s^-1^ gas hourly space velocity (GHSV).

| KIE | CD_3_CH(OH)CD_3_ | CH_3_CH(OD)CH_3_ |
| --- | --- | --- |
| IPA on (001) | 1.6 | 1 |
| H_2_O+ IPA on (001) | 1.9 | 1 |
| IPA on (101) | 1.7 | 1 |
| H_2_O+ IPA on (101) | 1.9 | 1 |

Table S5. Elementary Steps of IPA dehydration on TiO_2_(001)

| **Elementary Steps***^a^* | | **Parameters***^b^* |
| --- | --- | --- |
| (001)-1 | IPA (g) + Ti-O_s_-Ti(OH) 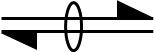 Ti(*i*OC_3_H_7_)-O_s_(H)-Ti(OH) | K_I,001_ |
| (001)-2 | Ti-O_s_-Ti(OH) + H_2_O (g) 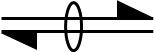 Ti(H_2_O)-O_s_-Ti(OH) | K_W1,001_ |
| (001)-3 | Ti(*i*-OC_3_H_7_)-O_s_(H)-Ti(OH) + H_2_O (g) 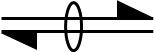 Ti(*i*-OC_3_H_7_···H_2_O)-O_s_(H)-Ti(OH) | K_W·D,001_ |
| (001)-4 | Ti(H_2_O)-O_s_-Ti(OH) + H_2_O (g) 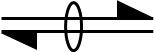 Ti(H_2_O)_2_-O_s_-Ti(OH) | K_W1-2,001_ |
| (001)-m-5 | Ti(*i*-OC_3_H_7_)-O_s_(H)-Ti(OH) 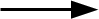 Ti(O)-O_s_(H)-Ti(H_2_O) + CH_2_CHCH_3_ (g) | k_m,001_ |
| (001)-m-6 | Ti(O)-O_s_(H)-Ti(H_2_O) 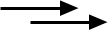 Ti-O_s_-Ti(OH) + H_2_O (g) |  |
| (001)-d-5 | Ti(*i*-OC_3_H_7_···H_2_O)-O_s_(H)-Ti(OH) 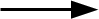Ti(O···H_2_O)-O_s_(H)-Ti(H_2_O) +CH_2_CHCH_3_ (g) | k_d,001_ |
| (001)-d-6 | Ti(O···H_2_O)-O_s_(H)-Ti(H_2_O) 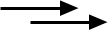 Ti-O_s_-Ti(OH) + 2H_2_O (g) |  |

*^a^*Arrow “
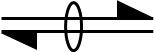
” represents a quasi-equilibrium reaction. “
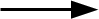
” represents an irreversible reaction. *^b^*K_I,001,_  K_W1,001_, K_W1-2,001_, K_W·D,001_ are the equilibrium constants corresponding to each elementary step, and k_m,001_ and k_d,001_ are the rate constants for **Steps (001)-m-5** and **(001)-d-5**, respectively.

Table S6. Elementary Steps of IPA dehydration on TiO_2_(101)

| **Elementary Steps***^a^* | | **Parameters***^b^* |
| --- | --- | --- |
| (101)-1 | IPA + Ti-O_s_ 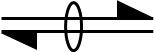 Ti(IPA)-O_s_ | K_I,101_ |
| (101)-2 | Ti-O_s_ + H_2_O (g) 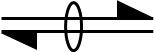 Ti(H_2_O)-O_s_ | K_W1,101_ |
| (101)-3 | Ti(IPA)-O_s_ + H_2_O (g) 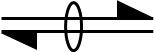 Ti(IPA···H_2_O)-O_s_ | K_W·D,101_ |
| (101)-4 | Ti(H_2_O)-O_s_ + H_2_O (g) 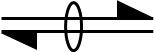 Ti(H_2_O)_2_-O_s_ | K_W1-2,101_ |
| (101)-m-5 | Ti(IPA)-O_s_ 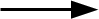 Ti(OH)-O_s_(H) +CH_2_CHCH_3_ (g) | k_m,101_ |
| (101)-m-6 | Ti(OH)-O_s_(H) 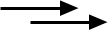 Ti-O_s_ + H_2_O (g) |  |
| (101)-d-5 | Ti(IPA···H_2_O)-O_s_ 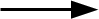 Ti(OH···H_2_O)-O_s_(H) +CH_2_CHCH_3_ (g) | k_d,101_ |
| (101)-d-6 | Ti(OH···H_2_O)-O_s_(H) 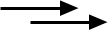 Ti-O_s_ + 2H_2_O (g) |  |

*^a^*Arrow “
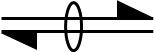
” represents a quasi-equilibrium reaction. “
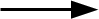
” represents an irreversible reaction. *^b^*K_I,101,_  K_W1,101_, K_W1-2,101_, K_W·D,101_ are the equilibrium constants corresponding to each elementary step, and k_m,101_ and k_d,101_ are the rate constants for **Steps (101)-m-5** and **(101)-d-5**, respectively.

Table S7. Kinetic and thermodynamic parameters obtained by the non-linear fitting of experimental data to rate expressions (eq. S33). Operating conditions: 260 °C, 1 atm total pressure with 0.25-8 kPa IPA, 0-8 kPa H_2_O, balance He, and 47.6 cm^3^ g^-1^ s^-1^ GHSV.

| Parameters | (001) | (101) |
| --- | --- | --- |
| k_d,j_ (s^-1^) | 2.49 (±0.15) × 10^-3^ | 7.10 (±0.90) × 10^-3^ |
| k_m,j_ (s^-1^) | 1.56 (±0.17) × 10^-2^ | 2.87 (±0.03) × 10^-2^ |
| K_I,j_ (Pa^-1^) | 1.29 (±0.10) × 10^-2^ | 1.73 (±0.01) × 10^-2^ |
| K_w·D,j_ (Pa^-1^) | 2.67 (±0.23) × 10^-3^ | 6.91 (±0.48) × 10^-4^ |
| K_w1,j_ (Pa^-1^) | 1.28 (±0.16) × 10^-2^ | 2.00 (±0.38) × 10^-3^ |
| K_w2,j_ (Pa^-2^) | 3.28 (±2.67) × 10^-7^ | 2.60 (±0.88) × 10^-7^ |

Table S8A. The fractional coverage of surface species and turnover frequencies of monomeric and complex pathway on TiO_2_(001) at 260 °C (calculated from eq. S33 and parameters in Table S7).

| Partial Pressure, Pa | | Fractional Coverages | | | | | r_d,001_, s^-1^ | r_m,001_, s^-1^ | r_d,001_/r_m,001_ |
| --- | --- | --- | --- | --- | --- | --- | --- | --- | --- |
| IPA | H_2_O | Ti-O_s_-Ti(OH) | Ti(*i*-OC_3_H_7_···  H_2_O)-O_s_(H)-Ti(OH) | Ti(H_2_O)-O_s_-Ti(OH) | (Ti(H_2_O)_2_-O_s_-Ti(OH) | Ti(*i*-OC_3_H_7_)-O_s_(H)-Ti(OH) |  |  |  |
| 250 | 0 | 24% | 0% | 0% | 0% | 76% | 0.000000 | 0.011880 | 0.00 |
| 500 | 0 | 13% | 0% | 0% | 0% | 87% | 0.000000 | 0.013476 | 0.00 |
| 1000 | 0 | 7% | 0% | 0% | 0% | 93% | 0.000000 | 0.014447 | 0.00 |
| 2000 | 0 | 4% | 0% | 0% | 0% | 96% | 0.000000 | 0.014987 | 0.00 |
| 4000 | 0 | 2% | 0% | 0% | 0% | 98% | 0.000000 | 0.015273 | 0.00 |
| 8000 | 0 | 1% | 0% | 0% | 0% | 99% | 0.000000 | 0.015419 | 0.00 |
| 250 | 2000 | 2% | 36% | 53% | 3% | 7% | 0.000886 | 0.001036 | 0.86 |
| 500 | 2000 | 1% | 50% | 37% | 2% | 9% | 0.001246 | 0.001457 | 0.86 |
| 1000 | 2000 | 1% | 63% | 23% | 1% | 12% | 0.001564 | 0.001829 | 0.86 |
| 2000 | 2000 | 1% | 72% | 13% | 1% | 13% | 0.001793 | 0.002096 | 0.86 |
| 4000 | 2000 | 0% | 78% | 7% | 0% | 15% | 0.001934 | 0.002262 | 0.86 |
| 8000 | 2000 | 0% | 81% | 4% | 0% | 15% | 0.002014 | 0.002355 | 0.86 |
| 250 | 4000 | 1% | 36% | 54% | 6% | 3% | 0.000901 | 0.000527 | 1.71 |
| 500 | 4000 | 1% | 52% | 39% | 4% | 5% | 0.001292 | 0.000755 | 1.71 |
| 1000 | 4000 | 0% | 66% | 25% | 3% | 6% | 0.001649 | 0.000964 | 1.71 |
| 2000 | 4000 | 0% | 77% | 14% | 1% | 7% | 0.001914 | 0.001119 | 1.71 |
| 4000 | 4000 | 0% | 83% | 8% | 1% | 8% | 0.002081 | 0.001216 | 1.71 |
| 8000 | 4000 | 0% | 87% | 4% | 0% | 8% | 0.002176 | 0.001272 | 1.71 |
| 250 | 8000 | 1% | 35% | 52% | 11% | 2% | 0.000872 | 0.000255 | 3.42 |
| 500 | 8000 | 0% | 51% | 38% | 8% | 2% | 0.001277 | 0.000373 | 3.42 |
| 1000 | 8000 | 0% | 67% | 25% | 5% | 3% | 0.001662 | 0.000486 | 3.42 |
| 2000 | 8000 | 0% | 79% | 15% | 3% | 4% | 0.001958 | 0.000572 | 3.42 |
| 4000 | 8000 | 0% | 86% | 8% | 2% | 4% | 0.002149 | 0.000628 | 3.42 |
| 8000 | 8000 | 0% | 91% | 4% | 1% | 4% | 0.002260 | 0.000660 | 3.42 |

Table S8B. The fractional coverages of surface species and turnover frequencies of monomeric and complex pathway on TiO_2_(101) at 260 °C (calculated from eq. S33 and parameters in Table S7).

| Partial Pressure, Pa | | Fractional Coverages | | | | | r_d,101_, s^-1^ | r_m,101_, s^-1^ | r_d,101_/  r_m,101_ |
| --- | --- | --- | --- | --- | --- | --- | --- | --- | --- |
| IPA | H_2_O | Ti-O_s_ | Ti(IPA···H_2_O)-O_s_ | Ti(H_2_O)-O_s_ | Ti(H_2_O)_2_-O_s_ | Ti(IPA)-O_s_ |  |  |  |
| 250 | 0 | 19% | 0% | 0% | 0% | 81% | 0.000000 | 0.023343 | 0.00 |
| 500 | 0 | 10% | 0% | 0% | 0% | 90% | 0.000000 | 0.025761 | 0.00 |
| 1000 | 0 | 5% | 0% | 0% | 0% | 95% | 0.000000 | 0.027168 | 0.00 |
| 2000 | 0 | 3% | 0% | 0% | 0% | 97% | 0.000000 | 0.027930 | 0.00 |
| 4000 | 0 | 1% | 0% | 0% | 0% | 99% | 0.000000 | 0.028328 | 0.00 |
| 8000 | 0 | 1% | 0% | 0% | 0% | 99% | 0.000000 | 0.028531 | 0.00 |
| 8000 | 0 | 1% | 0% | 0% | 0% | 99% | 0.000000 | 0.028531 | 0.00 |
| 250 | 2000 | 6% | 37% | 24% | 6% | 26% | 0.002597 | 0.007609 | 0.34 |
| 500 | 2000 | 4% | 45% | 15% | 4% | 32% | 0.003186 | 0.009334 | 0.34 |
| 1000 | 2000 | 2% | 51% | 8% | 2% | 37% | 0.003593 | 0.010526 | 0.34 |
| 2000 | 2000 | 1% | 54% | 5% | 1% | 39% | 0.003838 | 0.011245 | 0.34 |
| 4000 | 2000 | 1% | 56% | 2% | 1% | 41% | 0.003974 | 0.011642 | 0.34 |
| 8000 | 2000 | 0% | 57% | 1% | 0% | 41% | 0.004046 | 0.011852 | 0.34 |
| 250 | 4000 | 3% | 41% | 27% | 14% | 15% | 0.002884 | 0.004224 | 0.68 |
| 500 | 4000 | 2% | 52% | 17% | 9% | 19% | 0.003714 | 0.005440 | 0.68 |
| 1000 | 4000 | 1% | 61% | 10% | 5% | 22% | 0.004338 | 0.006354 | 0.68 |
| 2000 | 4000 | 1% | 67% | 6% | 3% | 24% | 0.004736 | 0.006938 | 0.68 |
| 4000 | 4000 | 0% | 70% | 3% | 2% | 25% | 0.004964 | 0.007271 | 0.68 |
| 8000 | 4000 | 0% | 72% | 1% | 1% | 26% | 0.005086 | 0.007450 | 0.68 |
| 250 | 8000 | 2% | 39% | 26% | 27% | 7% | 0.002744 | 0.002010 | 1.37 |
| 500 | 8000 | 1% | 53% | 18% | 18% | 10% | 0.003769 | 0.002760 | 1.37 |
| 1000 | 8000 | 1% | 65% | 11% | 11% | 12% | 0.004634 | 0.003394 | 1.37 |
| 2000 | 8000 | 0% | 74% | 6% | 6% | 13% | 0.005234 | 0.003834 | 1.37 |
| 4000 | 8000 | 0% | 79% | 3% | 3% | 14% | 0.005597 | 0.004099 | 1.37 |
| 8000 | 8000 | 0% | 82% | 2% | 2% | 15% | 0.005798 | 0.004247 | 1.37 |

**References:**

[62] Y. Wu, F. Gao, H. Wang, L. Kovarik, B. Sudduth, Y. Wang, *The Journal of Physical Chemistry C*  ***125***, (2021): 3988.

[63] X. Han, X. Wang, S. Xie, Q. Kuang, J. Ouyang, Z. Xie, L. Zheng, *RSC Advances*  ***2***, (2012).

[64] F. Lin, Y. Chen, L. Zhang, D. Mei, L. Kovarik, B. Sudduth, H. Wang, F. Gao, Y. Wang, *ACS Catalysis*  ***10***, (2020): 4268.

[65] N. R. Jaegers, W. Hu, Y. Wang, J. Z. Hu, *J. Vis. Exp.*, (2020).

[66] N. R. Jaegers, W. Hu, T. J. Weber, J. Z. Hu, *Scientific Reports*  ***11***, (2021): 7800.

[67] N. R. Jaegers, K. T. Mueller, Y. Wang, J. Z. Hu, *Acc Chem Res*  ***53***, (2020): 611.

[68] J. Z. Hu, M. Y. Hu, Z. Zhao, S. Xu, A. Vjunov, H. Shi, D. M. Camaioni, C. H. F. Peden, J. A. Lercher, *Chemical Communications*  ***51***, (2015): 13458.

[69] J. VandeVondele, M. Krack, F. Mohamed, M. Parrinello, T. Chassaing, J. Hutter, *Computer Physics Communications*  ***167***, (2005): 103.

[70] S. Goedecker, M. Teter, J. Hutter, *Physical Review B*  ***54***, (1996): 1703.

[71] J. P. Perdew, K. Burke, M. Ernzerhof, *Physical Review Letters*  ***77***, (1996): 3865.

[72] Z. Zhang, Y. Yoon, X. Lin, D. Acharya, B. D. Kay, R. Rousseau, Z. Dohnálek, *The Journal of Physical Chemistry Letters*  ***3***, (2012): 3257.

[73] S. Grimme, J. Antony, S. Ehrlich, H. Krieg, *The Journal of Chemical Physics*  ***132***, (2010): 154104.

[74] G. Henkelman, B. P. Uberuaga, H. Jónsson, *The Journal of Chemical Physics*  ***113***, (2000): 9901.

[75] N. R. Jaegers, J. K. Lai, Y. He, E. Walter, D. A. Dixon, M. Vasiliu, Y. Chen, C. Wang, M. Y. Hu, K. T. Mueller, I. E. Wachs, Y. Wang, J. Z. Hu, *Angew Chem Int Ed Engl*  ***58***, (2019): 12609.
